# Predictive-feedforward energy management for a hybrid agricultural machine operating in mountainous orchards

**DOI:** 10.1371/journal.pone.0360955

**Published:** 2026-10-16

**Authors:** Zuojin Li, Hao Huang, Linlu Dong, Xianfeng Zhang, Rui Zhou, Bo Li, Bao Yu

**Affiliations:** 1 College of Electronics and Electrical Engineering, Chongqing University of Science and Technology, Chongqing, China; 2 Institute of Agricultural Science and Technology Information, Chongqing Academy of Agricultural Sciences, Chongqing, China; National University of Singapore, SINGAPORE

## Abstract

Hybrid agricultural machines in mountainous orchards operate under low-speed, high-load conditions with coupled gradient and time-varying rolling-resistance disturbances. Their power demand is therefore nonstationary and may change abruptly. Rule-based and instantaneous strategies provide limited preview, whereas model predictive control (MPC) can transmit prediction errors directly to actuator commands and imposes substantial online computation. To address these limitations, a forward model was developed for a power-split hybrid agricultural machine comprising an engine, two electric machines, a traction battery, and a planetary gear set. A predictive-feedforward, multi-objective energy-management strategy was proposed. Short-horizon speed and gradient predictions are normalized, weighted, and saturated to form a bounded load-risk indicator. Rather than entering low-level torque calculation directly, this indicator modifies the dynamic state-of-charge (SOC) safety boundary, power-source mode, and transmission-ratio switching threshold, thereby softly decoupling prediction and execution. Engine high-efficiency operation, two transmission ratios, and actuator constraints are then coordinated for computationally efficient online power allocation. With offline dynamic programming (DP) as a common benchmark, the strategy achieved 93.29–95.59% of the DP performance over cycles C1–C3, exceeding MPC by 1.41–3.19 percentage points and adaptive equivalent-consumption minimization by 3.62–4.99 percentage points. Mean execution time was 0.128–0.191 ms per step, 93.54% lower than that of MPC, while terminal SOC remained near 0.600. No SOC, battery-current, or power-source torque constraint was violated under prediction noise or abrupt equivalent rolling-resistance disturbances. The conclusions apply to maintained, firm orchard lanes with low track slip; soft-soil operation involving high traction, severe sinkage, or substantial slip requires further validation. Bounded load-risk feedforward thus retained short-horizon preview while limiting direct error propagation, providing a practical real-time approach for hybrid agricultural machines with resource-constrained embedded controllers.

## Introduction

Orchards in hilly and mountainous regions often occupy slopes, terraces, and uneven terrain. Narrow lanes and frequent changes in gradient require agricultural machines to deliver high torque at low speed while remaining maneuverable and capable of climbing slopes. The adoption of machinery suited to these regions, lower-carbon agricultural practices, and autonomous orchard equipment has increased interest in coordinated power sources and intelligent control. In conventional engine-powered machines, repeated starts and stops, changing gradients, and fluctuating ground resistance can cause large shifts in engine operating points. Energy management must therefore account for tractive performance, fuel economy, battery charge balance, and real-time operation.

Previous reviews have classified the main hybrid-vehicle powertrain architectures [1]. Parallel and series–parallel configurations offer flexible power allocation through the coupling of engines, electric machines, and batteries [2,3]. Power-split architectures and multimode, multigear transmissions with clutches further extend the available coupling arrangements and operating modes [4,5]. Agricultural applications include modeling and hardware-in-the-loop evaluation of parallel hybrid tractors [6,7] and comparisons of hybrid architectures for specialized tractors [8]. An electrically continuously variable transmission (e-CVT) with power splitting can partly decouple engine speed from wheel-end load, while dual-motor coupling supports both operating-point regulation and propulsion [9,10]. Extended-range energy management, dynamic-programming-based powertrain optimization, and integrated power–thermal control have also been studied for hybrid tractors [11–13]. These studies provide a basis for agricultural hybrid-powertrain design. However, relatively few specifically address the combined effects of low-speed heavy loading, frequent starts and stops, and coupled gradient and time-varying rolling resistance in mountainous orchards. Their architectures and control rules require adaptation to these terrain conditions.

Rule-based energy management is straightforward to implement in real time, but empirically calibrated thresholds can lead to inefficient engine operation or state-of-charge (SOC) drift during abrupt load changes [14,15]. Instantaneous optimization has also been used to reduce hybrid-tractor energy consumption by allocating power according to the current operating state [16]; it does not, however, anticipate future changes. The equivalent consumption minimization strategy (ECMS) coordinates fuel and electrical energy instantaneously, but its equivalence factor is sensitive to engine transients, operating conditions, and SOC [17,18]. Dynamic programming (DP) provides a global optimum for a prescribed cycle and has been applied to agricultural hybrid tractors [19]. Improved solution methods and constraints on driving smoothness, SOC, and state of health have increased the computational efficiency and scope of DP evaluation [20,21]. Its dependence on complete cycle information nevertheless generally confines it to offline use. Model predictive control (MPC) uses future information in receding-horizon optimization, with performance that depends on prediction accuracy and online solution efficiency [22,23]. Combining ECMS with MPC improves adaptation to anticipated conditions but adds computational and implementation demands [24]. Related work has examined SOC constraints in predictive energy management and online tuning of ECMS equivalence factors [25,26]. Deep reinforcement learning has been applied to battery-health-aware energy management using naturalistic driving data [27], while intelligent coordination of hybrid energy storage has extended multisource control methods [28]. Training cost, reliability, and interpretability still require evaluation in practical applications. Energy-oriented torque allocation and hierarchical controllers that combine stability and energy conservation also show how multiple objectives can be coordinated at different control levels [29,30].

Prediction-assisted methods use historical information to anticipate operating changes. Long short-term memory (LSTM) networks have been used in hybrid-powertrain energy management [31], and rotary-tillage load prediction has supported coordinated control of unmanned hybrid tractors [32]. Road-vehicle studies have compared neural-network speed predictors [33] and incorporated LSTM-based multisignal speed prediction into MPC–ECMS [34]. Road preview has also supported eco-driving optimization of connected hybrid vehicles on sloped and curved roads [35]. Building on these studies, distributed and robust model predictive control and state-sensitive event-triggering have been applied to vehicle control to improve real-time performance and robustness under complex system constraints and resource limitations [36–38]. However, these studies focus primarily on speed prediction, implement-load prediction, road preview, or vehicle motion control; the joint representation of speed, gradient, and time-varying rolling resistance in mountainous agricultural operation remains less developed.

Three issues are particularly relevant to the predictive energy-management setting considered here. First, some controllers introduce predicted speed, gradient, or power demand directly into power or torque optimization, allowing prediction errors to propagate through the demand calculation to the actuators. Second, prediction-fusion weights are often fixed empirically without calibration and sensitivity analysis across cycles. Third, prediction and execution are not consistently separated through normalization, weighting, saturation, and current-state feedback. Predicted load also needs to be coordinated with dynamic SOC safety boundaries, efficient power-source operating regions, and transmission-ratio switching. The aim of this study was to retain short-horizon preview while limiting direct error propagation and maintaining a low computational burden.

We developed a forward model of a dual-motor power-split hybrid agricultural machine with gradient and time-varying rolling-resistance inputs, and designed a predictive-feedforward strategy for coordinated energy management. The contributions are threefold:

1. A forward model of a tracked machine with a dual-motor planetary power-split transmission represents low-speed heavy loading and coupled gradient–rolling-resistance disturbances. Kinematic and dynamic relationships are established for its operating modes.
2. A load-risk feedforward mechanism converts short-horizon speed and gradient predictions into a dimensionless indicator, limiting their direct transmission into power-source torque commands.
3. A power-allocation framework coordinates dynamic SOC safety boundaries, efficient engine operation, and two transmission ratios under physical actuator constraints. Offline DP provides a common performance reference for the online controller.

The following sections describe the architecture and forward model, present the controller and DP benchmark, and evaluate the complete strategy against online baselines. The evaluation includes ablation tests, computation time, robustness, dynamic response, and vehicle experiments, followed by conclusions and limitations.

## Vehicle design and specifications

### Hybrid powertrain architecture

Agricultural operations in hilly and mountainous terrain are characterized by low speeds, high torque demand, pronounced load fluctuations, and frequent gradient changes. Although a conventional mechanical driveline is simple and reliable, its engine operating point can vary substantially with terrain and equivalent tractive load, preventing sustained operation in a high-efficiency region. A hybrid agricultural machine was therefore designed, as illustrated schematically in Fig 1. Mechanical and electrical power paths are coupled so that the engine, electric machines, and battery can operate cooperatively according to the instantaneous task demand.

**Fig 1:**
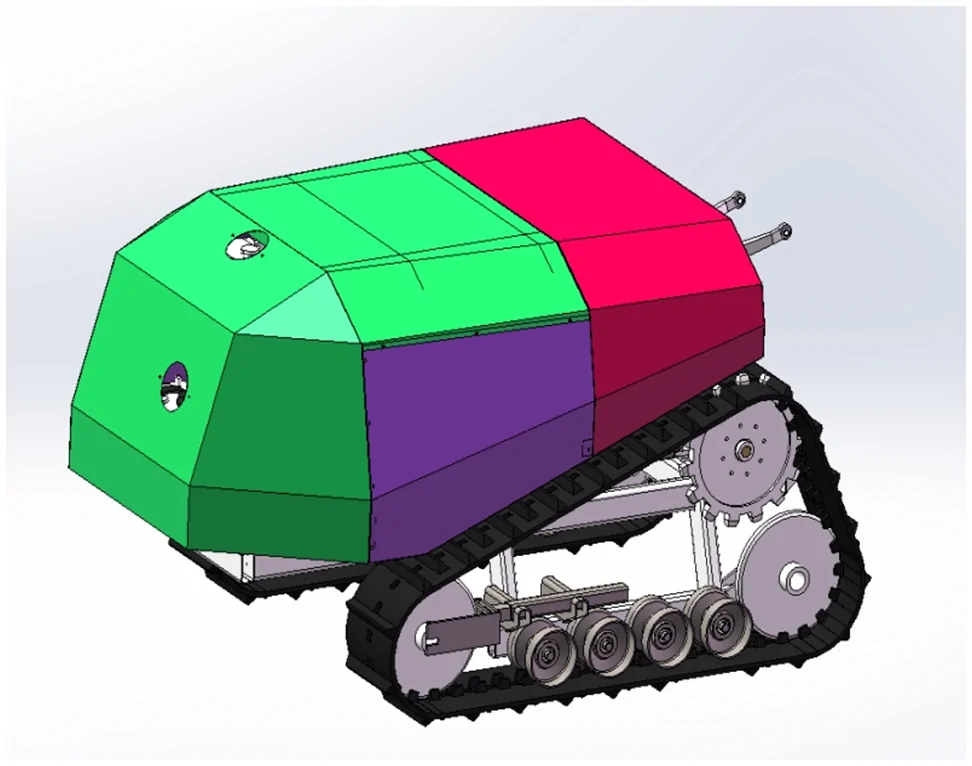
Schematic of the modular integrated design of the hybrid agricultural machine.

The architecture shown in Fig 2 consists of an engine, electric machines MG1 and MG2, a traction battery, a planetary power-coupling device, clutches C0 and C1, a reserved brake B1, a two-ratio transmission, and vehicle-dynamics modules. The engine supplies sustained operating power. MG1 is coupled to the planetary gear set and regulates engine speed, generates electrical power, and balances the power flow. MG2 is connected to the driveline output, supplies wheel-end torque, and recovers energy during downhill travel or braking. The battery buffers the power imbalance between the engine and the electric machines, supplies peak power, absorbs regenerative energy, and sustains SOC. The principal vehicle parameters are listed in Table 1. During launch, low-speed light-load operation, or brief low-power tasks, MG2 can propel the machine directly and prevent inefficient low-load engine operation. Under moderate-to-high loads or uphill travel, the engine and MG2 deliver power jointly while MG1 regulates the engine operating point. During deceleration or downhill travel, MG2 operates as a generator. When SOC is low, additional engine power is converted by MG1 to restore battery charge.

**Table 1:** Key vehicle parameters.

Component	Parameter	Value
Battery	Capacity	50 Ah
Battery	Rated voltage	388.8 V
Vehicle	Total mass	400 kg
Vehicle	Effective drive radius	0.25 m
Engine	Maximum speed	6000 r/min
Engine	Maximum torque	14.62 N·m
Drive motor (MG2)	Maximum speed	9000 r/min
Drive motor (MG2)	Maximum torque	27.69 N·m
Generator (MG1)	Maximum speed	9000 r/min
Generator (MG1)	Maximum torque	27.69 N·m
Planetary gear set	Characteristic ratio	2.6
Driveline	Transmission efficiency	0.95

**Fig 2:**
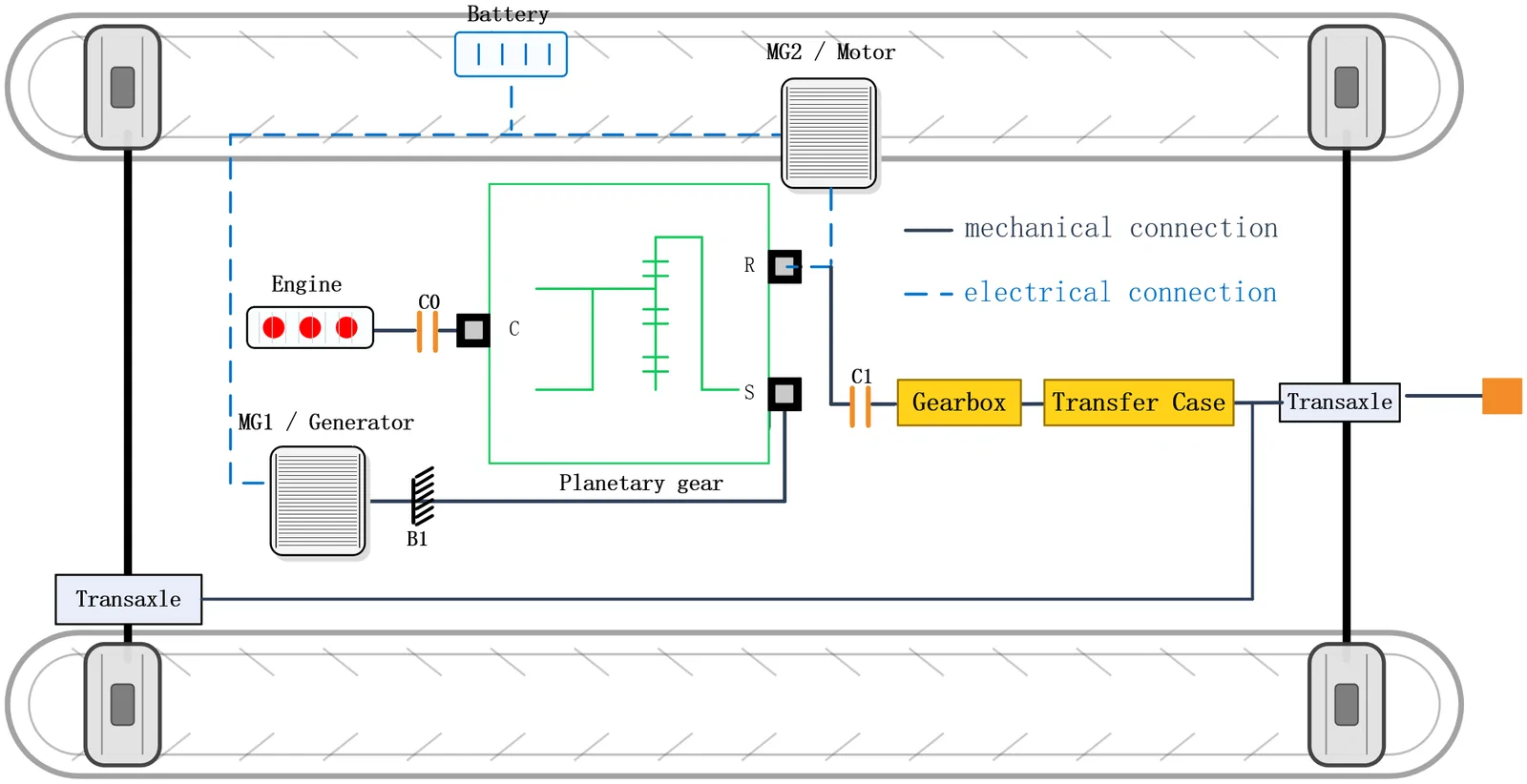
Powertrain architecture of the agricultural machine for mountainous terrain. The engine, MG1, and MG2 are coupled through a single planetary gear set and the downstream transmission.

The planetary power-coupling device enables multimode operation by imposing coupled speed and torque relationships among the engine, MG1, and MG2. Electric-machine control can therefore regulate engine speed while engine and motor power are distributed according to external load. The downstream transmission further adjusts wheel-end torque and vehicle speed to meet the requirements of low-speed field operation, grade travel, and terrain negotiation.

Based on power-source operating states, requested torque, vehicle speed, and battery SOC, the hybrid system was divided into several representative operating modes. Mode switching was determined jointly by operating load, battery charge, and braking demand rather than by vehicle speed alone, so that tractive performance, fuel economy, and charge balance could be maintained across operating conditions.

### Operating modes and kinematic characteristics

In the battery-electric mode, the engine is inactive and MG2 supplies the demanded drive torque. This mode is used for vehicle launch, low-speed light-load travel, and brief low-power operations, thereby exploiting the rapid low-speed torque response of the electric machine and avoiding high specific fuel consumption at low engine speed and load.

In the conventional hybrid mode, the engine, MG1, and MG2 operate cooperatively through the planetary gear set. The engine supplies most of the required power, MG1 regulates the engine operating point according to the planetary-speed relationship, and MG2 supplements the demanded torque. This mode is applied during sustained work, grade travel, and moderate-load operation.

In the charge-sustaining hybrid mode, engine output is increased beyond the instantaneous tractive demand and MG1 generates electrical power to recharge the battery. This mode is activated when SOC falls below its reference or after an extended high-load interval, thereby preventing progressive battery depletion.

During regenerative braking, the engine is shut down and MG2 converts part of the kinetic or gravitational potential energy into electrical energy. When the available regenerative torque is insufficient or SOC approaches its upper bound, the residual braking demand is supplied mechanically to preserve braking safety.

Collectively, the coordinated engine, dual-motor, battery, planetary-gear, and multimode transmission system provides battery-electric propulsion, hybrid propulsion, regenerative braking, and stationary charging. Table 2 summarizes the component states. The symbols P and N indicate positive- and negative-direction engine or motor operation, respectively. For clutches C0 and C1, E denotes engagement and D denotes disengagement. Vehicle holding is provided by a separate mechanical brake; B1 is reserved in the architecture and does not participate in mode switching.

**Table 2:** Power-component states in each operating mode.

Mode	Engine	MG1	MG2	C0	C1	B1
Vehicle holding	–	–	–	D	D	–
Battery-electric drive	–	–	P	D	E	–
Hybrid drive	P	N	P	E	E	–
Regenerative braking	–	–	N	D	E	–
Driving charge mode	P	N	P	E	E	–
Stationary charging	P	N	–	E	E	–

The engine, MG1, and mechanical output are connected to the carrier C, sun gear S, and ring gear R, respectively; MG2 torque is added to the mechanical output on the ring-gear side. The broad speed range of the sun gear enables MG1 to regulate engine speed and generate electrical power. Connecting the engine to the carrier divides engine power between the mechanical and electrical branches. The relatively low ring-gear speed and high torque capacity, together with MG2 torque assistance, are well suited to the low-speed, high-torque requirements of orchard machinery.

For the single planetary gear set in Fig 2, the tooth counts of the sun and ring gears are denoted by $Z_{S}$ and $Z_{R}$, respectively. Defining the characteristic ratio *k* as


$$\begin{array}{c} k=\frac{Z_{R}}{Z_{S}}, \end{array}$$ (1)

the speed constraint becomes


$$\begin{array}{c} \omega_{S}+k\omega_{R}=(1+k)\omega_{C}. \end{array}$$ (2)

When gear-meshing losses are neglected, the torque magnitudes and power balance satisfy


$$\begin{array}{c} |T_{S}|:|T_{R}|:|T_{C}|=1:k:(1+k), \end{array}$$ (3)

and


$$\begin{array}{c} P_{S}+P_{R}+P_{C}=0. \end{array}$$ (4)

Accordingly, engine input power is divided between the electrical branch containing MG1 and the mechanical output branch at the ring gear. MG1 changes the engine operating speed by controlling the sun-gear speed, while MG2 supplies traction assistance or regenerative braking at the output. In contrast to the rigid coupling between engine speed and wheel load in a fixed-ratio mechanical driveline, the power-split architecture provides an additional degree of freedom for shifting the engine operating point toward its high-efficiency region while satisfying low-speed, high-torque demand.

The mode-specific kinematic relationships are given below. The requested input torque and speed are denoted by $T_{req}$ and $n_{req}$. Engine torque and speed are $T_{e}$ and $n_{e}$; MG1 torque and speed are $T_{m1}$ and $n_{m1}$; and MG2 torque and speed are $T_{m2}$ and $n_{m2}$. The prescribed engine torque and speed during stationary charging are $T_{e,ch}$ and $n_{e,ch}$.

For vehicle holding,


$$\begin{array}{c} \left\{ \begin{array}{cc} T_{req} & =0,\\ n_{req} & =0,\\ T_{e} & =0,\\ n_{e} & =0,\\ T_{m1} & =0,\\ T_{m2} & =0,\\ n_{m1} & =0,\\ n_{m2} & =0. \end{array}\right. \end{array}$$ (5)

For battery-electric propulsion,


$$\begin{array}{c} \left\{ \begin{array}{cc} T_{e} & =0,\\ n_{e} & =0,\\ T_{m1} & =0,\\ T_{m2} & =T_{req},\\ n_{m2} & =n_{req},\\ n_{m1} & =-kn_{m2}. \end{array}\right. \end{array}$$ (6)

For conventional hybrid propulsion,


$$\begin{array}{c} \left\{ \begin{array}{cc} T_{e} & =T_{e}^{*},\\ n_{e} & =n_{e}^{*},\\ T_{m1} & =-\frac{T_{e}}{1+k},\\ T_{m2} & =T_{req}-\frac{kT_{e}}{1+k},\\ n_{m2} & =n_{req},\\ n_{m1} & =(1+k)n_{e}-kn_{m2}. \end{array}\right. \end{array}$$ (7)

For regenerative braking,


$$\begin{array}{c} \left\{ \begin{array}{cc} T_{e} & =0,\\ n_{e} & =0,\\ T_{m1} & =0,\\ T_{m2} & =T_{req},\\ n_{m2} & =n_{req},\\ n_{m1} & =-kn_{m2}. \end{array}\right. \end{array}$$ (8)

For stationary charging,


$$\begin{array}{c} \left\{ \begin{array}{cc} T_{req} & =0,\\ n_{req} & =0,\\ T_{e} & =T_{e,ch},\\ n_{e} & =n_{e,ch},\\ T_{m1} & =-\frac{T_{e}}{1+k},\\ T_{m2} & =0,\\ n_{m2} & =0,\\ n_{m1} & =(1+k)n_{e}. \end{array}\right. \end{array}$$ (9)

## Forward simulation model

### Engine model

The engine was represented by the brake-specific fuel-consumption (BSFC) map shown in Fig 3. Two-dimensional interpolation was used to obtain BSFC from engine speed and torque, after which mechanical power, fuel mass-flow rate, and cumulative fuel volume were calculated as follows:


$$\begin{array}{c} b_{e}=f(n_{e},T_{e}), \end{array}$$ (10)

$$\begin{array}{c} P_{e}=\frac{n_{e}T_{e}}{9549}, \end{array}$$ (11)

$$\begin{array}{c} {\dot{m}}_{f}=\frac{b_{e}P_{e}}{3600}, \end{array}$$ (12)

$$\begin{array}{c} V_{f}=\int_{0}^{t}\frac{{\dot{m}}_{f}}{\rho_{f}}\;dt. \end{array}$$ (13)

**Fig 3:**
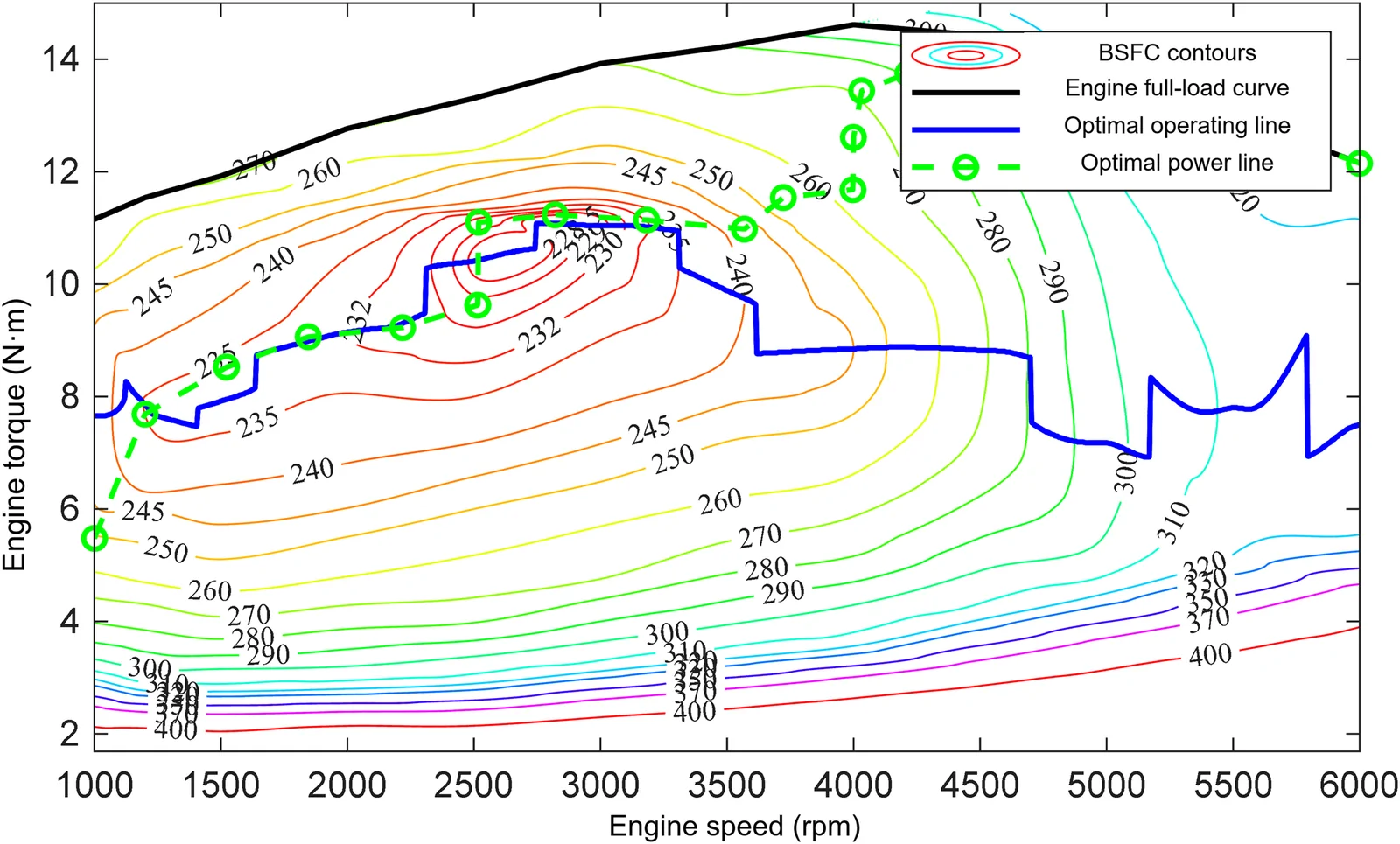
Engine brake-specific fuel-consumption map. Engine fuel consumption was obtained by two-dimensional interpolation over speed and torque.

Here, $b_{e}$ is the BSFC in g/(kW·h), $n_{e}$ and $T_{e}$ are the engine speed and torque, $P_{e}$ is the engine mechanical output power, ${\dot{m}}_{f}$ is the fuel mass-flow rate in g/s, $V_{f}$ is the cumulative fuel volume in L, and $\rho_{f}$ is the fuel density.

### Electric-machine model

Both MG1 and MG2 were modeled using the efficiency map shown in Fig 4. MG1 primarily generates electrical power and regulates the engine operating point, whereas MG2 provides traction and regenerative braking. Because efficiency varies substantially with speed and torque, two-dimensional map interpolation was used rather than a constant drive or generation efficiency. Electric-machine efficiency and mechanical power are expressed as


$$\begin{array}{c} \eta_{m}=f(n_{m},T_{m}), \end{array}$$ (14)

$$\begin{array}{c} P_{m}=\frac{n_{m}T_{m}}{9549}, \end{array}$$ (15)

where $\eta_{m}$, $n_{m}$, $T_{m}$, and $P_{m}$ denote efficiency, speed, torque, and mechanical power, respectively. Positive $P_{m}$ denotes motoring, whereas negative $P_{m}$ denotes generating or regenerative braking. The corresponding electrical power is


$$\begin{array}{c} P_{el}=\frac{P_{m}}{\eta_{m}},\;P_{m}\geq 0, \end{array}$$ (16)

$$\begin{array}{c} P_{el}=P_{m}\eta_{m},\;P_{m}<0. \end{array}$$ (17)

**Fig 4:**
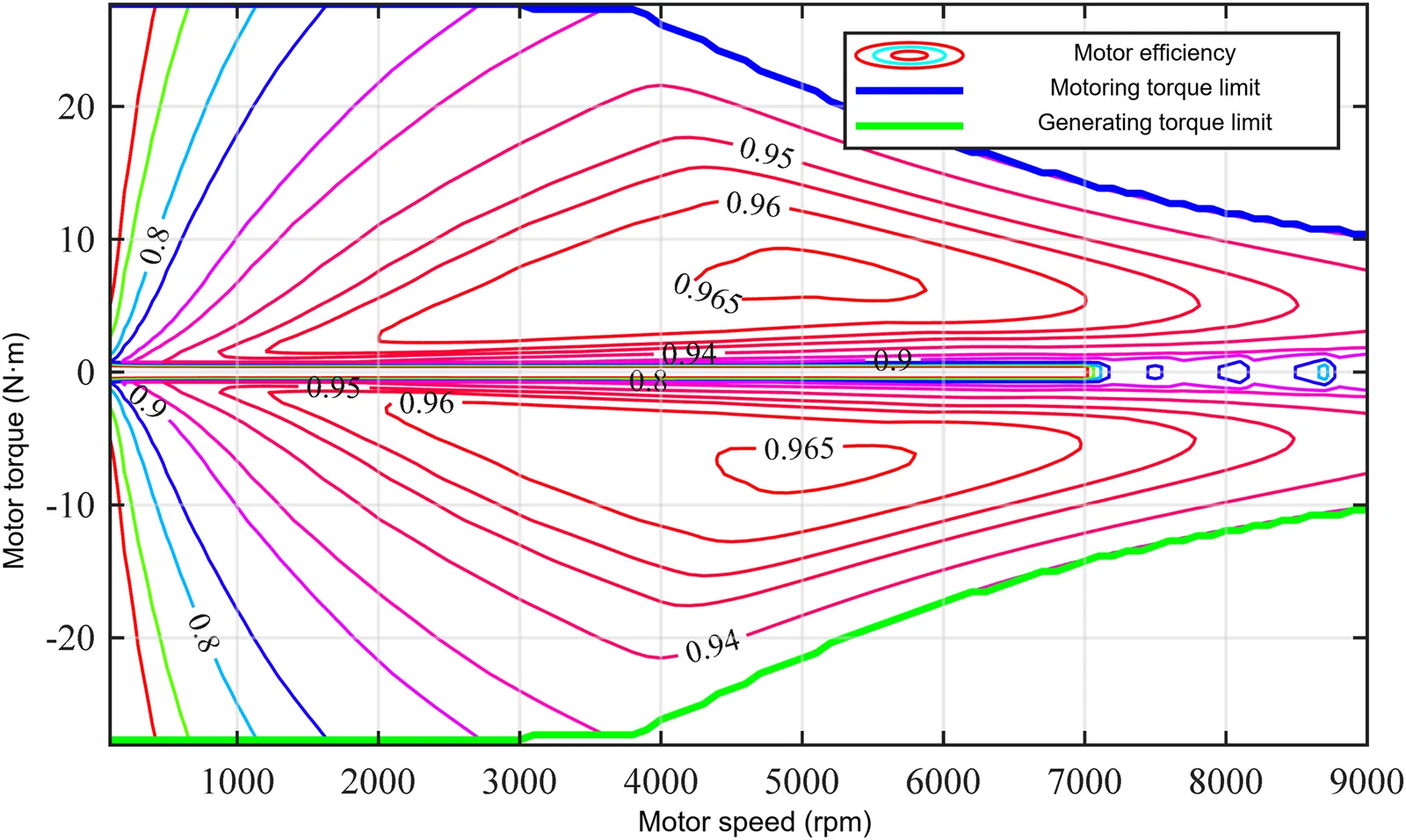
Electric-machine efficiency map. The same map-based formulation was applied to MG1 and MG2 according to their respective speed and torque.

Thus, battery output must compensate for conversion losses during motoring, and recoverable electrical power is reduced by the generating efficiency. The position of each motor operating point on the efficiency map therefore affects total equivalent energy consumption.

### Traction-battery model

The traction battery balances the power difference between the engine and electric machines and stores regenerated energy. The equivalent-circuit model in Fig 5 comprises an SOC-dependent open-circuit voltage source and a series resistance, thereby representing the effects of both open-circuit-voltage variation and internal-resistance voltage loss. Battery terminal voltage, power, and SOC dynamics are given by


$$\begin{array}{c} U_{bat}=V_{oc}(SOC)-I_{bat}R_{bat}, \end{array}$$ (18)

$$\begin{array}{c} P_{bat}=U_{bat}I_{bat}=V_{oc}I_{bat}-I_{bat}^{2}R_{bat}, \end{array}$$ (19)

$$\begin{array}{c} SOC(t)=SOC(t_{0})-\frac{1}{3600Q_{bat}}\int_{t_{0}}^{t}I_{bat}\;dt. \end{array}$$ (20)

**Fig 5:**
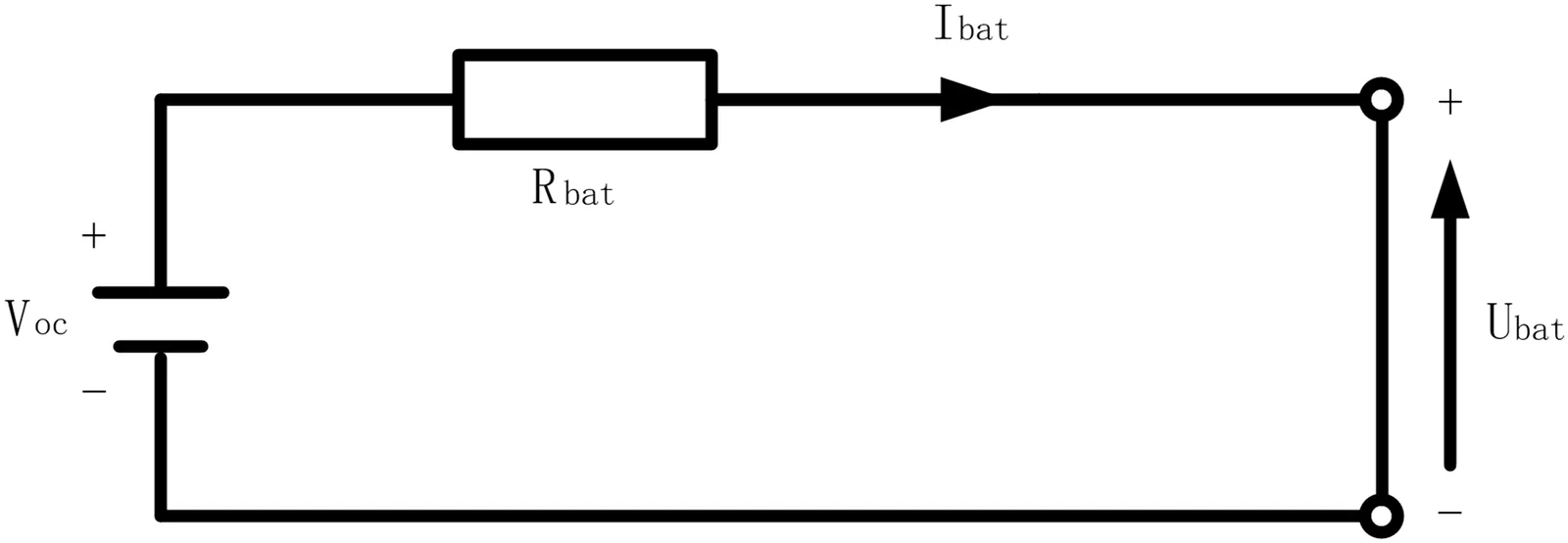
Equivalent internal-resistance model of the traction battery. Open-circuit voltage varies with SOC and the series resistance produces the load-dependent terminal-voltage drop.

Here, $U_{bat}$ is terminal voltage, $V_{oc}$ is open-circuit voltage, $I_{bat}$ is battery current, $R_{bat}$ is the equivalent pack resistance, $P_{bat}$ is battery power, and $Q_{bat}$ is nominal capacity. Discharge current is defined as positive; consequently, discharge decreases SOC, whereas charging and regeneration increase SOC. The pack contains 108 cells connected in series, giving a rated voltage of 388.8 V without increasing the 50-Ah cell capacity. Near *SOC* = 0.6, the pack open-circuit voltage is approximately 406.08 V, so an SOC change of 0.001 corresponds to approximately 20.3 Wh.

### Transmission and external-load model

The transmission converts power-source torque into wheel-end tractive force and determines the MG2 speed at a given vehicle speed. For mountainous agricultural operation, the selected ratio affects both wheel torque and the location of the MG2 operating point on its efficiency map. The transmission relationships are


$$\begin{array}{c} T_{w}=\eta_{t}i_{g}T_{in}, \end{array}$$ (21)

$$\begin{array}{c} n_{m2}=\frac{vi_{g}}{0.377r_{w}}, \end{array}$$ (22)

where $T_{w}$ is wheel-end drive torque, $T_{in}$ is transmission input torque, $\eta_{t}$ is transmission efficiency, $i_{g}$ is the transmission ratio, *v* is vehicle speed in km/h, and $r_{w}$ is the effective drive-sprocket radius.

Gradient and ground resistance vary more strongly in mountainous agricultural operations than in conventional road driving. The required longitudinal force and transmission-input torque were therefore calculated as


$$\begin{array}{c} F_{t}=m\frac{dv_{SI}}{dt}+mg\sin\theta +mg\mu \cos\theta , \end{array}$$ (23)

$$\begin{array}{c} T_{req}=\frac{F_{t}r_{w}}{\eta_{t}i_{g}}, \end{array}$$ (24)

where $v_{SI}=v/3.6$ is speed in m/s for the acceleration term, $F_{t}$ is required longitudinal force, *m* is total mass, *g* is gravitational acceleration, θ is the road-gradient angle, μ is the rollin*g*-resistance coefficient, and $T_{req}$ is the torque requested at the transmission input. The formulation converts terrain gradient and surface-resistance variation into a real-time load boundary for energy-consumption calculations.

The architecture and controller were developed for maintained orchard lanes that are comparatively smooth, firm, and characterized by continuously varying longitudinal gradients and low track slip. Ground-condition variation is represented by an equivalent rolling-resistance coefficient. Track sinkage, substantial slip, soil shear deformation, and nonlinear slip–traction coupling in soft soil were not modeled. Accordingly, the present configuration and control conclusions should not be transferred directly to soft cropland, deeply tilled soil, or sustained high-draft operation. Such applications require a coupled soil–track model and renewed calibration of both powertrain parameters and energy-management boundaries.

On the basis of the preceding component connections and operating modes, the forward MATLAB/Simulink model shown in Fig 6 was constructed to represent the coupling among driver demand, power-source output, the transmission, and vehicle motion. Component connections and kinematic constraints define the physical boundaries, while nonlinear engine, electric-machine, battery, and external-load behavior captures changes in energy consumption caused by gradient, rolling resistance, and component efficiency.

**Fig 6:**
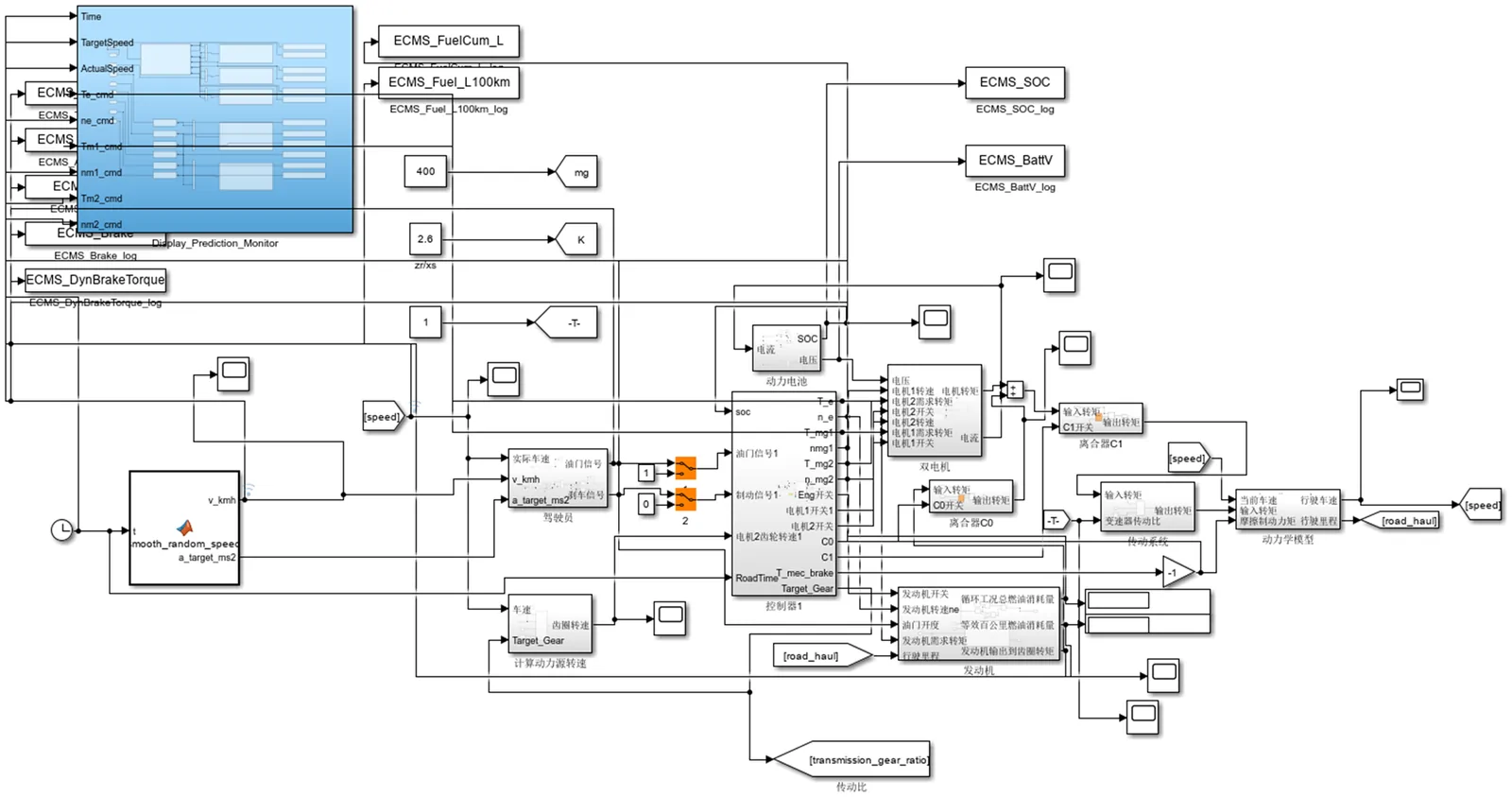
Overview of the forward simulation model. The MATLAB/Simulink model couples driver demand, power sources, battery, transmission, and longitudinal vehicle dynamics.

### Predictive-feedforward multi-objective energy management

Unlike predictive MPC, in which future speed and gradient sequences enter an online optimizer that directly calculates power-source torque, the proposed controller first transforms the short-horizon predictions into a bounded load-risk indicator. This indicator modifies the dynamic SOC safety boundary, power-source mode, and transmission-ratio switching threshold. Torque is subsequently allocated among the engine and two electric machines under the planetary-gear constraints. The resulting hierarchy comprises short-horizon prediction, risk-indicator construction, SOC and mode coordination, transmission-ratio selection, torque allocation, and physical-boundary verification. Prediction does not enter low-level torque calculation directly, thereby limiting direct error propagation to the actuators.

### Online prediction and load-risk indicator

Short-horizon prediction was based on local continuity of the maintained orchard lanes considered in this study. Their longitudinal gradients vary continuously and predominantly at low frequency over the distance scale of agricultural operation, whereas soil moisture, compaction, and local surface condition produce faster changes in rolling resistance. Historical windows were therefore used to extrapolate speed and gradient, while the rolling-resistance coefficient was treated as the principal time-varying disturbance. This assumption restricts the controller to maintained orchard lanes; untreated terrain containing steps, ditches, or abrupt gradient discontinuities requires further validation.

Separate historical windows were formed for speed *v* and gradient θ, each containing *N* = 10 samples at 1-s intervals. Locally Gaussian-weighted quadratic regression was used to extrapolate the state over the next 5 s. Let $\varphi (x)=[1,x,x^{2}{]}^{T}$ and let *W* be the local Gaussian weight matrix. The regression coefficients and the prediction *h* seconds ahead are


$$\begin{array}{c} \beta_{y}=(\Phi^{T}W\Phi +\lambda I{)}^{-1}\Phi^{T}Wy, \end{array}$$ (25)

$$\begin{array}{c} \hat{y}(t+h|t)=\varphi^{T}(h)\beta_{y}+\frac{h}{5}e_{y}(t). \end{array}$$ (26)

Here, y∈{v,θ} and h∈[0,5] s; the controller uses the endpoint prediction at *h* = 5 s. The design matrix Φ is assembled from the historical basis functions, the Gaussian bandwidth is σ=4, and the regularization coefficient is $\lambda =10^{-6}$. The online residual $e_{y}(t)$ is updated with a forgetting factor of 0.86.

The predicted endpoint speed $\hat{v}$ and gradient $\hat{\theta }$ are fused with the normalized observed load $l_{obs}$ to form the dimensionless load-risk indicator


$$\begin{array}{c} L_{p}={\mathrm{sat}}_{\left[0,1\right]}\left(0.64l_{obs}+0.18s_{v}+0.30s_{up}-0.12s_{down}\right), \end{array}$$ (27)

where


$$\begin{array}{cc} s_{v} & ={\mathrm{sat}}_{\left[0,1\right]}\left(\frac{\hat{v}}{9}\right),\\ s_{up} & ={\mathrm{sat}}_{\left[0,1\right]}\left(\frac{\hat{\theta }}{28}\right),\\ s_{down} & ={\mathrm{sat}}_{\left[0,1\right]}\left(\frac{-\hat{\theta }}{14}\right). \end{array}$$ (28)

The saturation operator ${\mathrm{sat}}_{\left[0,1\right]}(\cdot )$ limits its argument to [0,1]. The terms $s_{v}$, $s_{up}$, and $s_{down}$ are normalized predictions of speed, uphill gradient, and downhill gradient, respectively. Because the current load directly represents instantaneous power demand, $l_{obs}$ receives the largest weight. Speed and uphill predictions contribute positively, whereas downhill gradient provides a negative correction.

The weights were tested around the nominal combination (0.64,0.18,0.30,−0.12) at 80%, 100%, and 120% of their nominal values. An $L_{9}(3^{4})$ orthogonal design was evaluated over cycles C1–C3, comprising nine orthogonal combinations and one nominal combination per cycle, for a total of 30 simulations. The main-effect range of DP attainment rate is shown in Fig 7. The range associated with the observed-load weight was 0.268–1.229 percentage points and exceeded that of every prediction weight, whose range did not exceed 0.243 percentage points. The difference between the nominal combination and the best sampled combination was 0.027, 0.307, and 0.612 percentage points for C1, C2, and C3, respectively.

**Fig 7:**
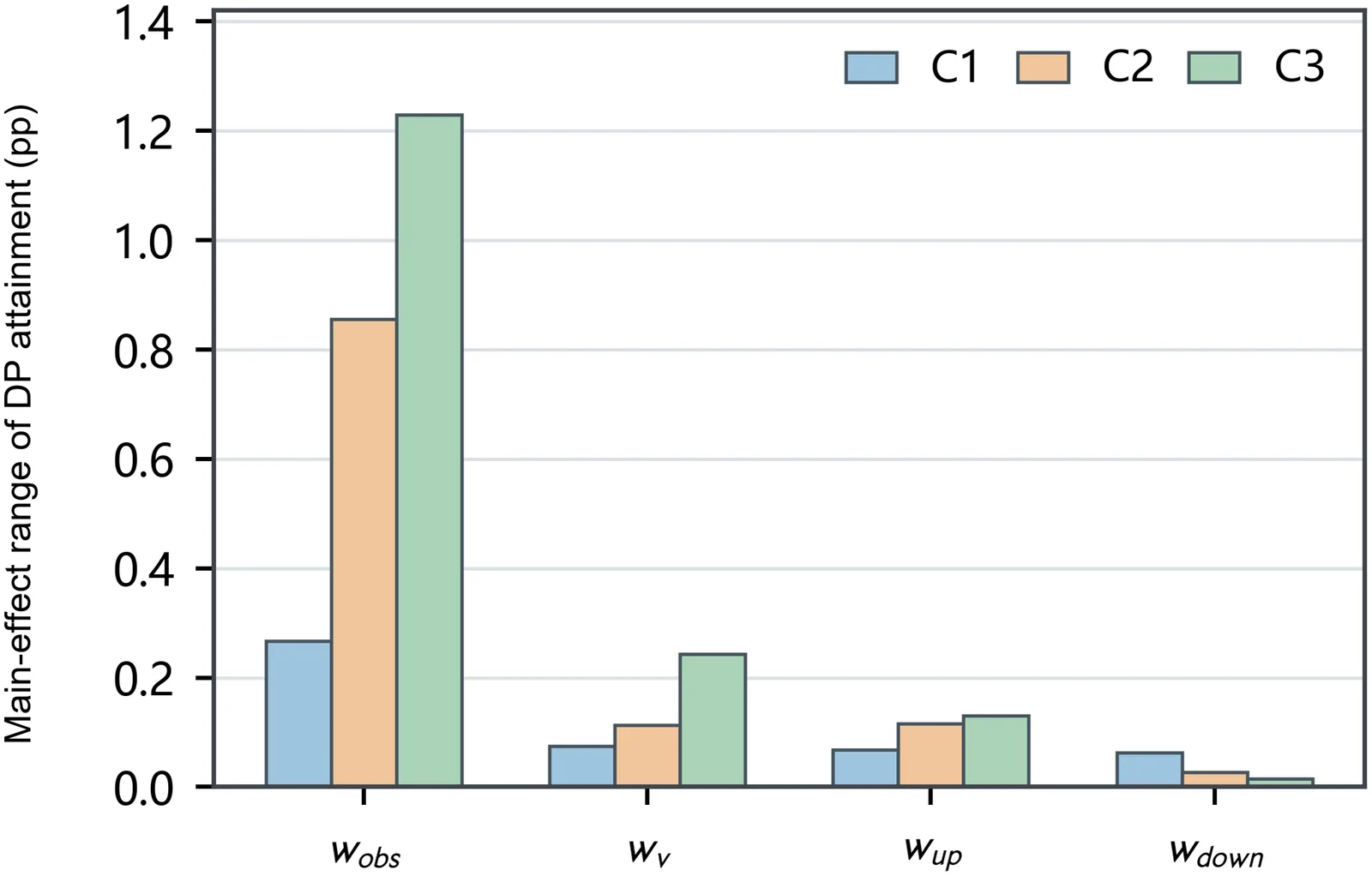
Sensitivity of the load-risk weights. Main-effect ranges of DP attainment rate for the four load-risk weights over cycles C1, C2, and C3.

Because saturation is a nonexpansive mapping, the contributions of prediction error to the load-risk indicator are bounded by


$$\begin{array}{cc} \varepsilon_{v} & =\frac{0.18}{9}|\Delta \hat{v}|,\\ \varepsilon_{\theta +} & =\frac{0.30}{28}|\Delta {\hat{\theta }}_{+}|,\\ \varepsilon_{\theta -} & =\frac{0.12}{14}|\Delta {\hat{\theta }}_{-}|, \end{array}$$ (29)

and


$$\begin{array}{c} |\Delta L_{p}|\leq \varepsilon_{v}+\varepsilon_{\theta +}+\varepsilon_{\theta -}. \end{array}$$ (30)

The quantities $\Delta \hat{v}$, $\Delta {\hat{\theta }}_{+}$, and $\Delta {\hat{\theta }}_{-}$ denote prediction-error components for speed, uphill gradient, and downhill gradient, and the corresponding ε terms denote their weighted upper-bound contributions. Prediction errors therefore affect mode and operating-point decisions only after normalization, weighting, and saturation. Even when saturation is active, $L_{p}$ remains bounded within [0,1], reducing the risk that prediction errors are amplified directly into power-source torque commands.

The propagation from load risk to actuator commands can be considered further by writing the torque-command vector as $u=[T_{e},T_{m1},T_{m2}{]}^{T}$. With operating mode, transmission ratio, and the active constraint set fixed, the downstream law consists of affine operations, saturation, and minimum/maximum limits. It is therefore piecewise Lipschitz, with $\left\|\Delta u\|\leq L_{u,l}|\Delta L_{p}|+L_{u,x}\|\Delta x\right\|$, where *x* contains feedback states such as actual requested torque, speed, and SOC. Near mode or ratio switching surfaces, the holding logic and actuator constraints still keep commands bounded. This architecture does not eliminate prediction error; it replaces its direct role in low-level torque calculation with a bounded supervisory variable, limiting propagation to the execution layer.

### State-dependent power-source coordination

The power-source mode is selected from the load-risk indicator, requested torque, and SOC. When predicted load is low and SOC is above its target interval, MG2 is preferentially used to meet the tractive demand, thereby avoiding low-load, low-efficiency engine operation. As predicted load increases or SOC approaches a safety boundary, the engine is activated. Its target torque is selected jointly from requested torque and the efficient engine operating region. If engine output exceeds the immediate tractive demand, MG1 absorbs the surplus power and charges the battery.

The system is operated in a charge-sustaining manner with both initial and reference SOC set to 0.600. Bidirectional closed-loop correction is applied around this reference. When SOC exceeds its prescribed interval, battery discharge and electric-drive participation are increased. When SOC falls below the interval, the engine and MG1 generate additional electrical energy. The objective is therefore bidirectional correction toward the reference rather than enforcement of a lower SOC bound alone, enabling sustainable battery-energy balance over the operating cycle while meeting total power demand.

### SOC–transmission coordination and power allocation

To accommodate both sustained load migration caused by gradual gradient variation and transient disturbances caused by start–stop operation or changes in ground resistance, the lower SOC boundary is adjusted according to predicted load:


$$\begin{array}{c} SOC_{min}^{*}=\mathrm{sat}\left(SOC_{min}+k^{T}s_{SOC}\right), \end{array}$$ (31)

The transmission switches between ratios 32 and 40. The larger ratio is selected under high load or elevated SOC risk to move MG2 toward a more efficient operating region; otherwise, the baseline ratio is retained:


$$\begin{array}{c} i_{g}^{des}=32+8R_{g},\;R_{g}\in \{0,1\}. \end{array}$$ (32)

Once engine target torque and transmission ratio have been determined, MG1 and MG2 commands are calculated from the planetary torque relationships. Engine torque and speed, motor torque and speed, and battery SOC are checked against their physical limits. Saturation and a safe candidate state are used whenever a requested command is infeasible. For negative requested torque, MG2 regenerative braking is prioritized. As SOC approaches its upper bound, regenerative torque is limited and the remaining demand is supplied by the mechanical brake.

Conflicting objectives are handled hierarchically. Safety and physical feasibility have the highest priority, followed by meeting requested wheel torque, maintaining SOC balance, and improving fuel economy and ratio-switching smoothness.

### Control workflow and offline benchmark

The complete workflow is summarized in Fig 8. Online prediction, load-risk feedforward, SOC supervision, power-source coordination, transmission-ratio selection, and physical-boundary checks form a closed supervisory loop. Offline DP is not used to generate online commands.

**Fig 8:**
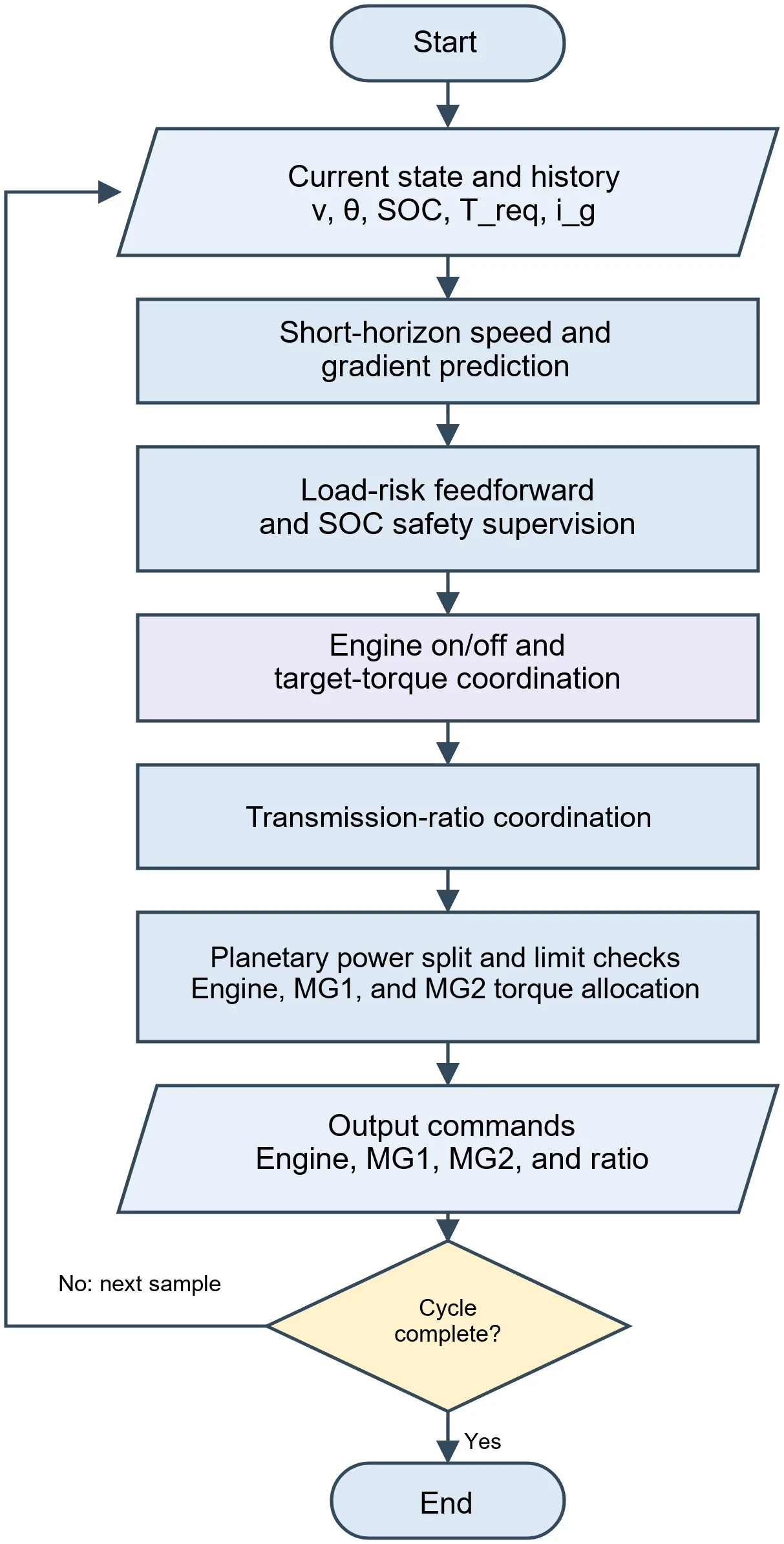
Workflow of the predictive-feedforward multi-objective energy-management strategy. Predicted speed and gradient are converted into a bounded supervisory indicator before mode, transmission-ratio, and torque decisions are made.

A consistent offline DP benchmark was constructed using the same vehicle parameters, efficiency maps, battery model, and transmission-ratio candidates as the online simulation, thereby providing a cycle-specific offline performance reference. Based on Bellman’s principle of optimality,


$$\begin{array}{c} V_{k}(x)=\min_{u}\left[g_{k}(x,u)+V_{k+1}(x^{+})\right]. \end{array}$$ (33)

The state is $x_{k}=[SOC_{k},i_{g,k-1}{]}^{T}$ and the control is $u_{k}=[T_{e,k},i_{g,k}{]}^{T}$, where $i_{g,k-1}$ and $i_{g,k}$ are the previous and current transmission ratios. Each 600-s operating cycle was discretized into 300 stages with Δt=2 s. The SOC grid extended from 0.560 to 0.620 with $\Delta SOC=5\times 10^{-4}$, and the candidate ratio set was $i_{g}\in \{32,40\}$.

The DP discretization was assessed by one-factor-at-a-time grid refinement over C1–C3. The nominal increments were $\Delta SOC=5\times 10^{-4}$, $\Delta T_{e}=0.25$ N·m, and Δt=2 s. The tested values were $\Delta SOC\in \{2\times 10^{-3},10^{-3},5\times 10^{-4},2.5\times 10^{-4},1.25\times 10^{-4}\}$, $\Delta T_{e}\in \{1,0.5,0.25,0.125,0.0625,0.03125,0.015625\}$ N·m, and Δt∈{8,4,2,1,0.5} s. All other inputs, component parameters, constraints, and terminal-SOC corrections were held fixed, and the three scans shared the nominal setting.

Relative to the finest grid in each scan, the largest deviation in corrected fuel consumption across the SOC grids was 0.000425%. The corresponding maxima across the torque and time grids were 0.364885% and 0.464517%; the nominal settings differed from their respective finest grids by at most 0.178307% and 0.079781%. The changes between the final two refinement levels decreased further for torque and time discretization in all three cycles, while the SOC-grid results were nearly unchanged. The largest absolute terminal-SOC difference between the nominal setting and any corresponding finest grid was $4.71\times 10^{-4}$. No requested-torque truncation occurred during replay of the optimal policies. Fig 9 shows that corrected fuel use and terminal SOC were only weakly sensitive to further refinement within the tested range. The nominal discretization thus provided an engineering compromise between numerical accuracy and computation.

**Fig 9:**
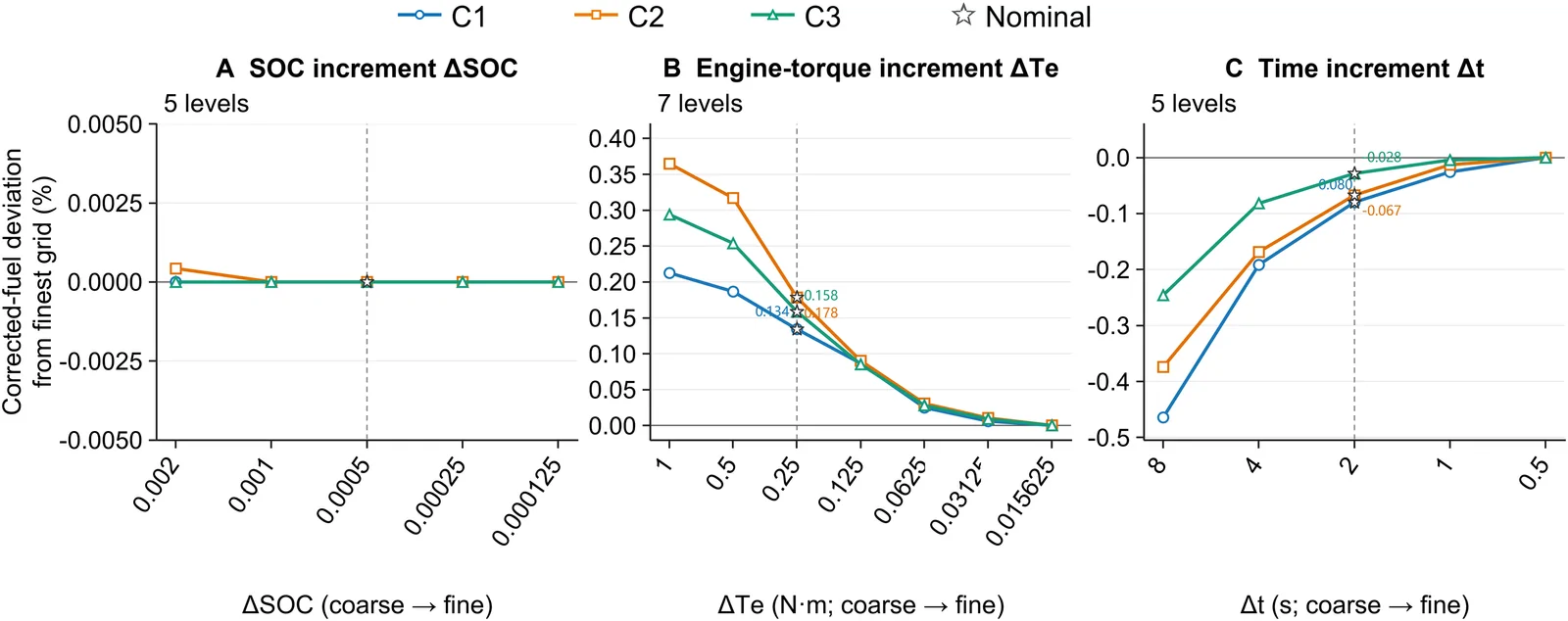
Grid convergence of DP state, control, and time discretization. Relative changes in SOC-corrected fuel consumption are referenced to the finest grid in each one-factor scan. Panels A–C show SOC, engine-torque, and time increments, respectively.

DP first returned the actual fuel consumption and terminal SOC over each cycle. During post-processing, the same terminal-SOC energy correction used for the online controllers was applied. Within a cycle, DP and all online strategies used identical $SOC_{ref}$ and $K_{SOC}$. The coefficient was determined offline before controller comparison; it was neither used by online control nor fitted to the outcome of an individual strategy. This procedure imposed a common terminal-energy basis and prevented unequal residual battery energy from distorting the fuel-economy comparison.

## Results and discussion

The predictive-feedforward and multi-objective coordination strategy was evaluated over three representative mountainous agricultural operating cycles. All controllers used identical powertrain parameters, engine and electric-machine efficiency maps, battery model, transmission-ratio candidates, and terminal-SOC evaluation criteria. Performance was assessed using terminal-SOC-corrected fuel consumption, terminal-SOC consistency, DP attainment rate, ablation results, computational cost, robustness, and power-source operating behavior. Offline DP served only as a cycle-specific performance reference and did not participate in online control. Simulations were performed on a custom MATLAB/Simulink platform using normalized external reference data. The host computer contained an Intel Core i5-14490F processor, an NVIDIA GeForce RTX 5060 8-GB graphics processor, and 32 GB of memory.

### Operating cycles and evaluation metrics

All three cycles were restricted to low-speed agricultural operation below 10 km/h. C1 emphasized start–stop operation, changing gradients, and abrupt rolling-resistance changes; C2 emphasized continuous travel and gradually varying gradients; and C3 combined speed steps with composite gradient variation. Their characteristics are summarized in Table 3, and the associated target-speed and gradient profiles are shown in Fig 10. Continuous, gradually varying gradient curves were used to represent the longitudinal profiles of maintained orchard lanes. Rapid disturbances caused by soil and surface condition were introduced through the time-varying rolling-resistance coefficient, retaining the complex load variation relevant to the target environment.

**Table 3:** Characteristics of the three simulated operating cycles.

Cycle	Duration (s)	Mean speed (km/h)	Max. speed (km/h)	Stopped (%)	Gradient (°)	Rolling resistance
C1	600	5.81	9.82	12.33	−2.29–13.78	0.103–0.148
C2	600	5.35	7.43	2.33	2.42–9.66	0.124–0.151
C3	600	5.93	8.30	0.00	−1.92–12.04	0.100–0.137

**Fig 10:**
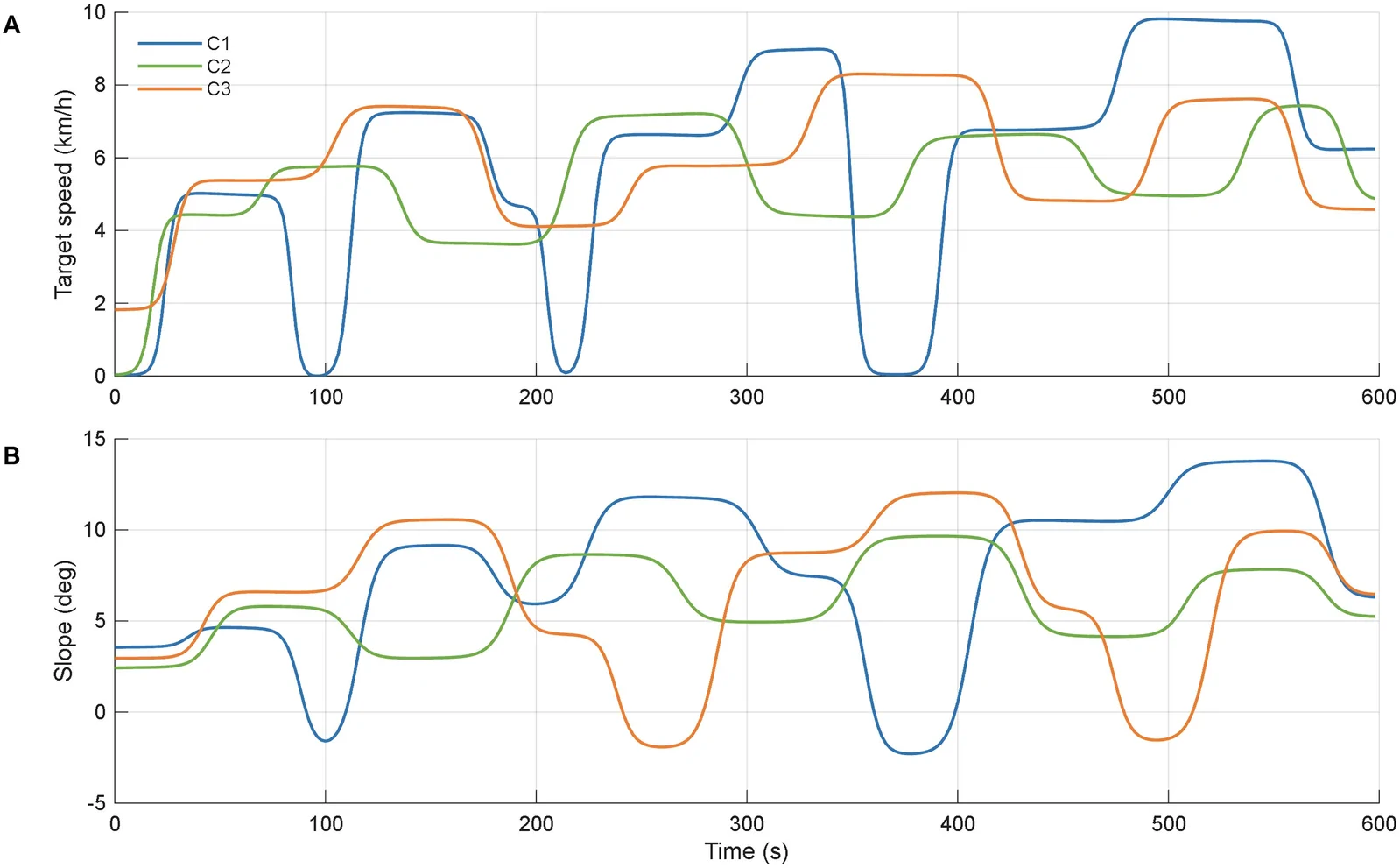
Target-speed and road-gradient profiles for cycles C1–C3. Panels A and B show target speed and road gradient, respectively, for the three cycles.

Actual fuel consumption was obtained from the cumulative fuel volume over each 600-s simulation. Because apparent fuel savings in a charge-sustaining hybrid system can be distorted by unequal terminal battery energy, the terminal energy difference was converted into equivalent fuel using a common fuel-to-storage conversion efficiency. SOC-corrected fuel consumption was therefore used to evaluate actual fuel use and terminal charge sustenance on a common basis. The SOC-corrected fuel consumption and DP attainment rate were defined as


$$\begin{array}{c} m_{corr}=m_{f}+K_{SOC}(SOC_{ref}-SOC_{f}), \end{array}$$ (34)

$$\begin{array}{c} R_{DP}=\frac{m_{DP,corr}}{m_{corr}}\times 100\%. \end{array}$$ (35)

where $m_{corr}$ is SOC-corrected fuel consumption, $m_{f}$ is actual fuel consumption, $SOC_{f}$ is terminal SOC, $SOC_{ref}$ is reference SOC, and $m_{DP,corr}$ is the SOC-corrected offline DP result obtained under the same cycle and constraints.

The correction coefficient $K_{SOC}$ was determined from physically feasible charging operating points. Requested torque and power-source speeds were calculated from target speed, gradient, and rolling resistance. At each valid time step, the incremental fuel power of the best charging point relative to the best noncharging point was used to estimate fuel-to-electrical-energy conversion efficiency. The median of the optimal valid efficiencies was adopted as the cycle charging efficiency, producing $K_{SOC}=5257.3553293317$ g/SOC:


$$\begin{array}{c} K_{SOC}=\frac{Q_{bat}\;3600\;V_{oc}(SOC_{ref})}{H_{u}\eta_{c}}. \end{array}$$ (36)

The actual DP outputs are listed in Table 4. For every online controller, $R_{DP}$ was calculated using the corrected DP fuel value from the same cycle and the same terminal-SOC correction.

**Table 4:** Actual outputs of the offline DP benchmark.

Cycle	Actual fuel (g)	Corrected fuel (g)	Initial SOC	Terminal SOC
C1	114.389	96.062	0.600000	0.603486
C2	123.343	74.473	0.600000	0.609295
C3	124.484	79.482	0.600000	0.608560

### Overall performance of the complete strategy

The complete strategy produced corrected fuel-consumption values of 102.974, 78.272, and 83.152 g in C1, C2, and C3, respectively, corresponding to DP attainment rates of 93.29%, 95.15%, and 95.59% (Table 5). Terminal SOC remained close to 0.600 in all three cycles, indicating that the apparent fuel economy was not obtained through substantial net battery depletion. Eq (34) applies a signed correction: corrected consumption increases when terminal SOC is below its reference and decreases when residual battery energy exceeds the reference. Controllers are thereby compared at a common terminal-energy basis. The SOC trajectories are shown in Fig 11.

**Table 5:** Fuel economy and SOC balance of FULL.

Cycle	Fuel (g)	Corrected fuel (g)	Initial SOC	Terminal SOC	ΔSOC	$R_{DP}$ (%)
C1	98.041	102.974	0.6000	0.599062	−0.000938	93.29
C2	77.274	78.272	0.6000	0.599810	−0.000190	95.15
C3	81.735	83.152	0.6000	0.599731	−0.000269	95.59

**Fig 11:**
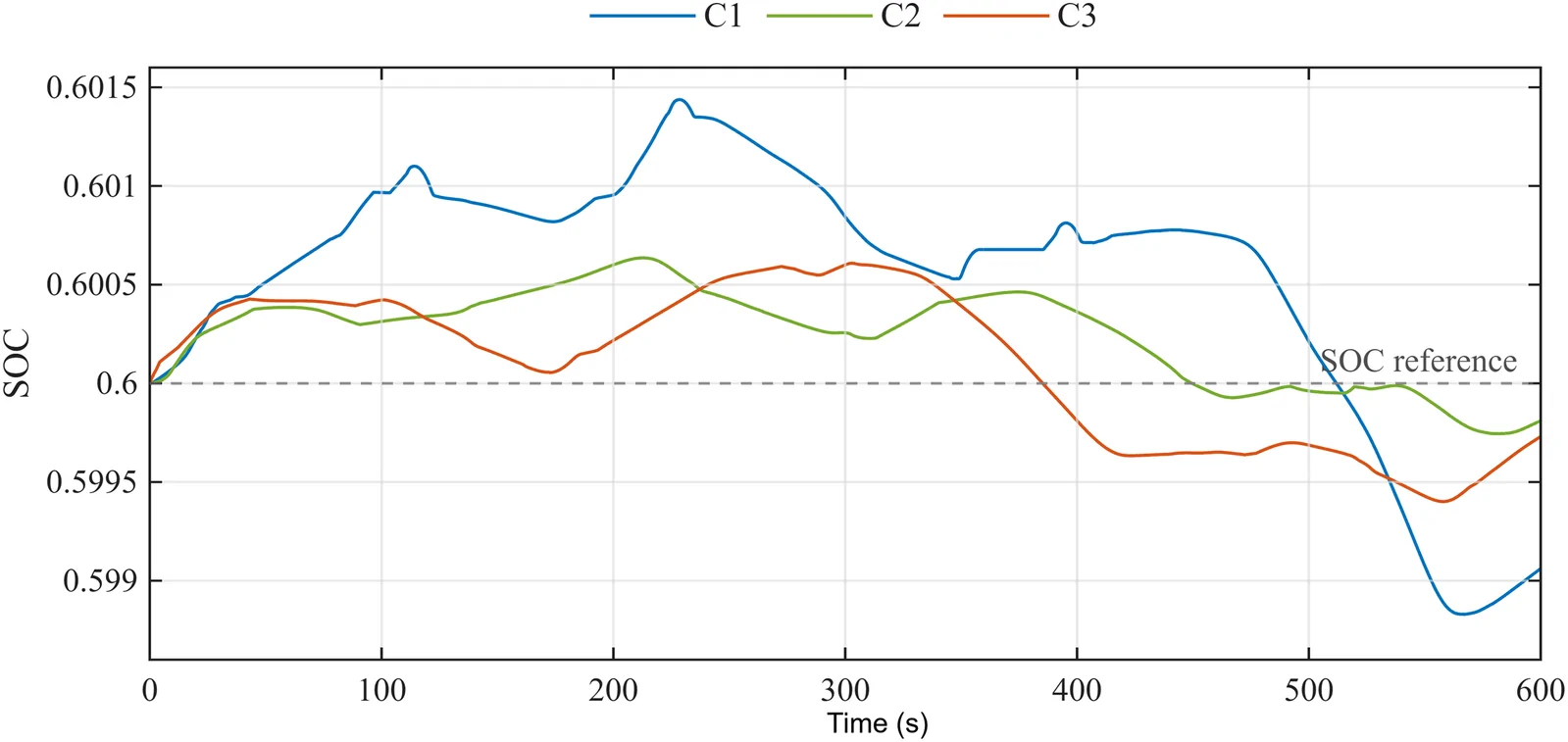
SOC trajectories of the complete strategy over cycles C1–C3. C1 exhibits larger short-duration fluctuations because of its start–stop, gradient, and rolling-resistance disturbances, whereas C2 and C3 vary more gradually.

Although the C1 SOC trajectory fluctuated more visibly during start–stop intervals and load transitions, it remained within a narrow band over the full cycle. The smoother C2 and C3 trajectories further indicate that the proposed controller sustained battery energy under different load patterns and provided a consistent basis for corrected-fuel comparison.

### Comparison with online baseline strategies

MPC, adaptive ECMS (A-ECMS), and a rule-based strategy were used as online baselines. All four controllers shared the same vehicle model, component parameters, physical actuator limits, cycle inputs, and SOC evaluation procedure. Identical $SOC_{ref}$ and $K_{SOC}$ values were used within each cycle; differences in corrected fuel therefore arose only from actual fuel use and terminal-SOC deviation. MPC used finite-horizon receding optimization, A-ECMS adjusted its equivalence factor online from SOC error, and the rule-based baseline applied common thresholds to engine start–stop and power allocation. To prevent differences in upper-level safety constraints from confounding the comparison, all controllers shared actuator limits and SOC safety supervision. A-ECMS should therefore be interpreted as a matched baseline with shared safety supervision rather than as a completely independent standard implementation. Results are presented in Fig 12 and Table 6.

**Table 6:** Comparison of FULL with online baseline strategies.

Cycle	Strategy	Actual fuel (L)	Corrected fuel (g)	Terminal SOC	$R_{DP}$ (%)
C1	FULL	0.135415	102.974	0.599062	93.29
C1	MPC	0.081438	106.614	0.590936	90.10
C1	A-ECMS	0.112351	108.789	0.594779	88.30
C1	Rule based	0.095432	116.655	0.590953	82.35
C2	FULL	0.106732	78.272	0.599810	95.15
C2	MPC	0.082329	80.085	0.596105	92.99
C2	A-ECMS	0.119202	81.366	0.600939	91.53
C2	Rule based	0.103098	89.284	0.597215	83.41
C3	FULL	0.112894	83.152	0.599731	95.59
C3	MPC	0.097638	84.395	0.597393	94.18
C3	A-ECMS	0.118839	87.018	0.599814	91.34
C3	Rule based	0.104162	94.565	0.596357	84.05

**Fig 12:**
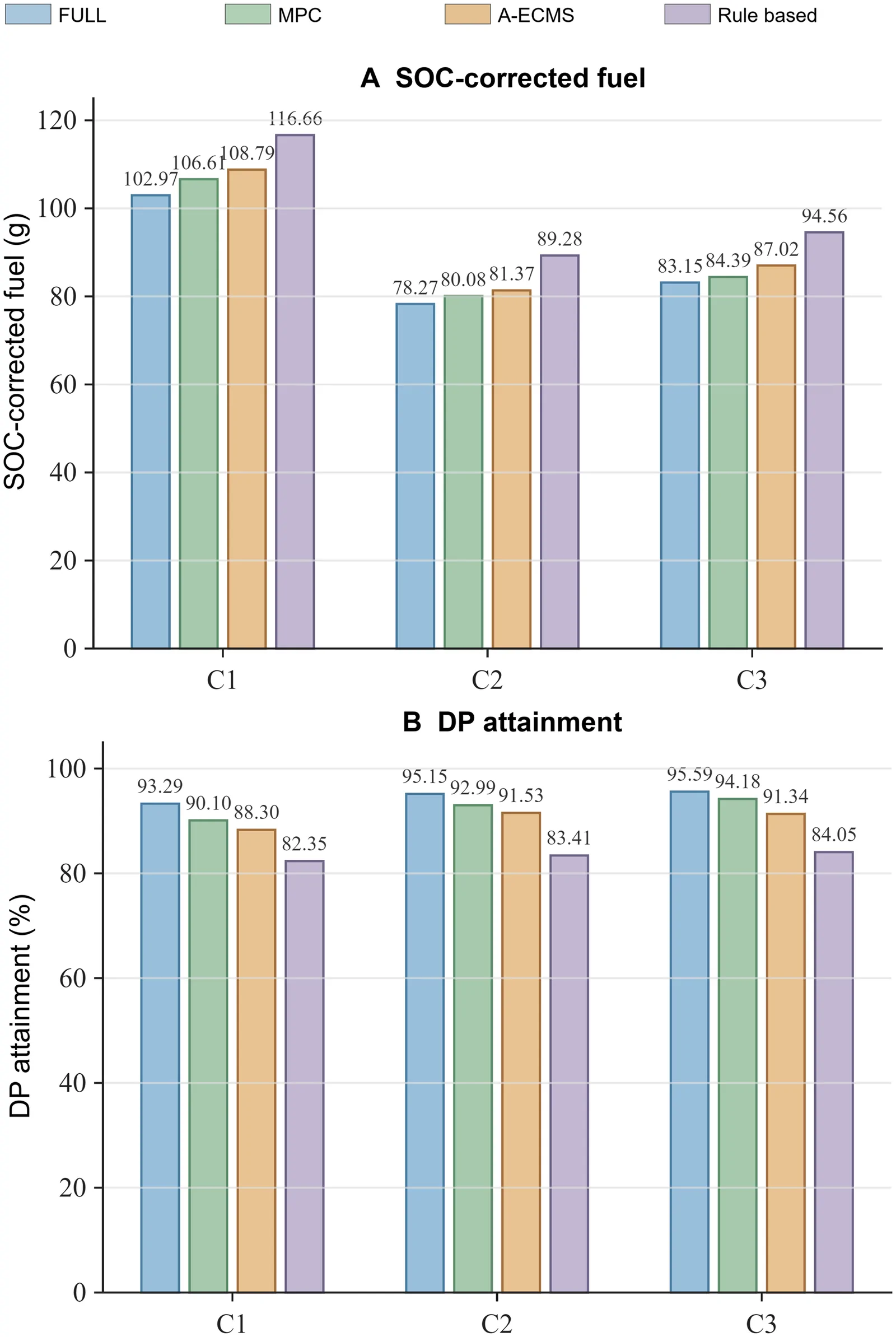
SOC-corrected fuel consumption and relative DP performance of the online energy-management strategies. FULL denotes the complete predictive-feedforward strategy.

Relative to MPC, FULL increased the DP attainment rate by 3.19, 2.16, and 1.41 percentage points in C1, C2, and C3, respectively. The corresponding gains over A-ECMS were 4.99, 3.62, and 4.25 percentage points, while those over the rule-based baseline were 10.94, 11.74, and 11.54 percentage points. This ordering was consistent across all cycles. Short-horizon speed–gradient risk feedforward, coordinated SOC supervision, and transmission-ratio selection therefore compensated for the limited preview of instantaneous strategies without changing the evaluation boundary.

### Control-module ablation

To distinguish the contributions of prediction, transmission-ratio coordination, and dynamic SOC boundaries, we evaluated variants without speed prediction, gradient prediction, either predictive channel, or dynamic SOC-boundary adjustment, together with fixed-ratio variants at 32 and 40. Each variant used FULL as its reference and disabled only the channel under examination or fixed the corresponding control variable. Component parameters, cycle inputs, power-source coordination rules, and corrected-fuel evaluation were otherwise unchanged. The fixed-ratio variants did not switch ratios. Removing the dynamic SOC boundary retained the static safety limits and removed only their prediction-dependent offsets. Fig 13 shows the decrease in DP attainment relative to the corresponding FULL reference; Table 7 reports corrected fuel use, terminal SOC, attainment, and its decrease.

**Table 7:** Control-module ablation results.

Cycle	Variant	Corrected fuel (g)	Terminal SOC	$R_{DP}$ (%)	Attainment loss (pp)
C1	FULL	102.974	0.599062	93.29	0.00
C1	No speed prediction	111.730	0.592880	85.98	7.31
C1	No gradient prediction	107.550	0.594372	89.32	3.97
C1	No speed/gradient prediction	114.139	0.591391	84.16	9.13
C1	Fixed ratio 32	104.217	0.599636	92.18	1.11
C1	Fixed ratio 40	103.192	0.598246	93.09	0.20
C1	FULL, low SOC	102.770	0.593707	93.45	0.00
C1	No dynamic SOC boundary	102.805	0.593655	93.42	0.03
C2	FULL	78.272	0.599810	95.15	0.00
C2	No speed prediction	80.784	0.601034	92.19	2.96
C2	No gradient prediction	79.333	0.600684	93.87	1.28
C2	No speed/gradient prediction	83.806	0.599242	88.86	6.29
C2	Fixed ratio 32	78.468	0.603427	94.91	0.24
C2	Fixed ratio 40	78.855	0.599348	94.44	0.70
C2	FULL, low SOC	77.553	0.594813	95.95	0.00
C2	No dynamic SOC boundary	77.646	0.594190	95.84	0.12
C3	FULL	83.152	0.599731	95.59	0.00
C3	No speed prediction	86.251	0.599856	92.15	3.44
C3	No gradient prediction	88.840	0.598803	89.47	6.12
C3	No speed/gradient prediction	94.233	0.596126	84.35	11.24
C3	Fixed ratio 32	83.623	0.601071	95.05	0.54
C3	Fixed ratio 40	83.649	0.599155	95.02	0.57
C3	FULL, low SOC	82.051	0.595506	96.80	0.00
C3	No dynamic SOC boundary	82.461	0.594560	96.32	0.48

*Note:* pp, percentage points. Low-SOC FULL and its dynamic-boundary ablation start at SOC = 0.594 and use a DP reference recalculated at that initial SOC. Other variants start at SOC = 0.600. Losses are relative to the corresponding FULL reference.

**Fig 13:**
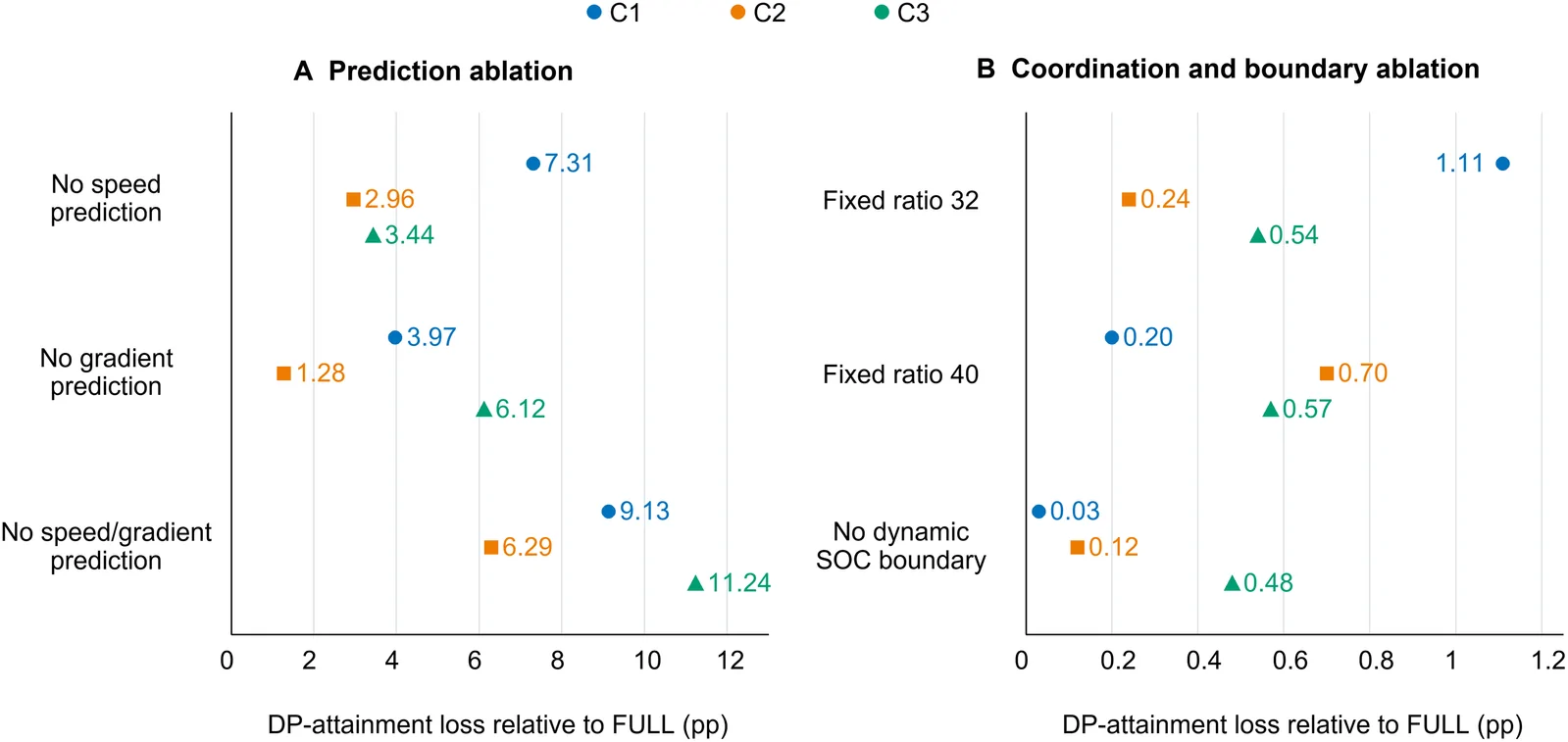
DP-attainment losses after control-module ablation. Panels A and B show prediction-channel ablations and coordination/boundary ablations, respectively. Dynamic SOC-boundary ablation is compared with FULL initialized at SOC = 0.594; the other ablations use SOC = 0.600.

The effects depended on the dominant load characteristics of each cycle. Removing prediction had the largest effect. Without speed prediction, DP attainment was 85.98% in C1 and 92.19% in C2, decreases of 7.31 and 2.96 percentage points, respectively. These losses exceeded those without gradient prediction, at 3.97 and 1.28 percentage points. Speed preview thus had a larger influence on energy allocation during starts, stops, acceleration, deceleration, and sustained load migration in these cycles. In C3, removing gradient prediction reduced attainment to 89.47%, a loss of 6.12 percentage points, compared with 3.44 percentage points without speed prediction. Gradient information was therefore more influential when speed steps coincided with composite gradient changes. Removing both channels reduced attainment to 84.16%, 88.86%, and 84.35% in C1–C3, corresponding to losses of 9.13, 6.29, and 11.24 percentage points. The two sources of preview information made complementary contributions to load-trend identification and power-source coordination.

Disabling ratio coordination had a smaller effect than removing prediction, but reduced performance in all three cycles. At a fixed ratio of 32, attainment was 92.18%, 94.91%, and 95.05%, with losses of 1.11, 0.24, and 0.54 percentage points. At a fixed ratio of 40, the respective losses were 0.20, 0.70, and 0.57 percentage points. Switching between 32 and 40 allowed operating points to be adjusted to different load ranges and improved performance across cycles.

Dynamic SOC-boundary adjustment is activated by proximity to a constraint and was rarely triggered from the usual initial SOC of 0.600. A separate low-SOC group was therefore initialized at 0.594, with FULL and DP recalculated for each cycle at that same initial state. Apart from the dynamic-boundary channel, inputs, control parameters, and evaluation methods were identical. Removing the dynamic boundary reduced DP attainment by 0.033, 0.115, and 0.481 percentage points in C1–C3. In C1, cumulative time below SOC = 0.5935 increased from 14.14 to 25.27 s. Terminal SOC fell from 0.594813 to 0.594190 in C2 and from 0.595506 to 0.594560 in C3. The main role of this module was therefore to restrict further energy depletion near the SOC boundary, improving constraint protection rather than substantially reducing average fuel use under nominal conditions.

### Computational performance

The online computational burden was measured over 600-s simulations of FULL and receding-horizon MPC using an Intel Core i5-14490F processor and MATLAB/Simulink R2025a. Both used Normal simulation mode, a fixed step of 0.01 s, and identical vehicle models, cycle inputs, and signal logging. As shown in Fig 14, total simulation times for FULL were 30.20, 18.78, and 17.97 s in C1–C3; MPC required 163.53, 171.21, and 170.26 s, or 5.42–9.47 times as long. Excluding initialization and termination, mean execution time per step was 0.128–0.191 ms for FULL and 2.271–2.402 ms for MPC. The execution-stage computation time was 93.54% lower for FULL.

**Fig 14:**
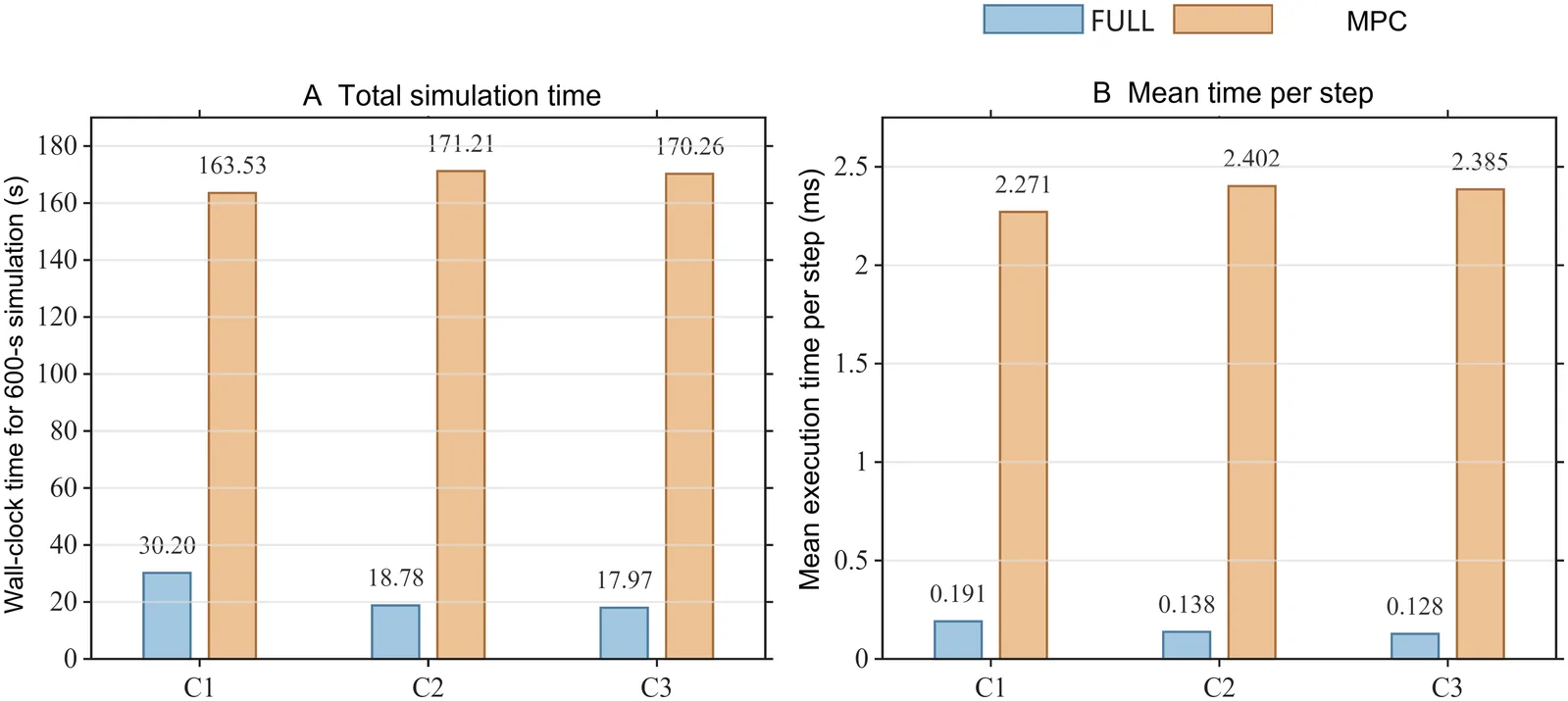
Simulation and per-step execution times for FULL and MPC. Panel A shows total wall-clock time for each 600-s simulation. Panel B shows mean execution time per 0.01-s step, excluding initialization and termination.

Orchard machinery is used seasonally and often has limited annual utilization, making purchase and maintenance costs consequential. Meeting real-time requirements through more powerful processors also increases controller, cooling, and power-supply costs. FULL avoids the repeated online search over finite-horizon candidate sequences required by MPC. Its reduced computation, together with the observed fuel economy and SOC regulation, is relevant to the cost, reliability, and maintainability requirements of agricultural controllers.

### Robustness

Prediction errors and abrupt additional ground loads were tested over 600 s in C1–C3. Colored random noise with root-mean-square (RMS) amplitudes of 0.5, 1.0, 1.5, and 2.0 km/h was added to predicted speed, and amplitudes of 0.5°, 1.0°, 1.5°, and 2.0° were added to predicted gradient. Speed-only, gradient-only, and combined disturbances were evaluated with 10 random seeds at each level. Equivalent rolling-resistance increments of +0.12 and +0.16 were applied at 268–276 s and 452–460 s, respectively, to represent brief additional resistance from local gravel cover or loose surface material.

With the rolling-resistance-only case as the reference, the four prediction-noise levels produced DP-attainment changes ranging from −0.0273 to 0.0341 percentage points (Fig 15). The small negative values in C2 reflect fluctuations in the random-sample means and do not establish a beneficial effect of noise. The two rolling-resistance events reduced attainment by 1.0700, 1.4239, and 0.9650 percentage points in C1–C3. No SOC, battery-current, engine-torque, or motor-torque constraint was violated in the tested scenarios. Compared with a matched variant that omitted the observed-load term, FULL retained absolute attainment advantages of 0.459, 0.593, and 0.901 percentage points under the rolling-resistance disturbances. These results support its performance under the tested disturbances, without establishing suppression of arbitrary prediction errors.

**Fig 15:**
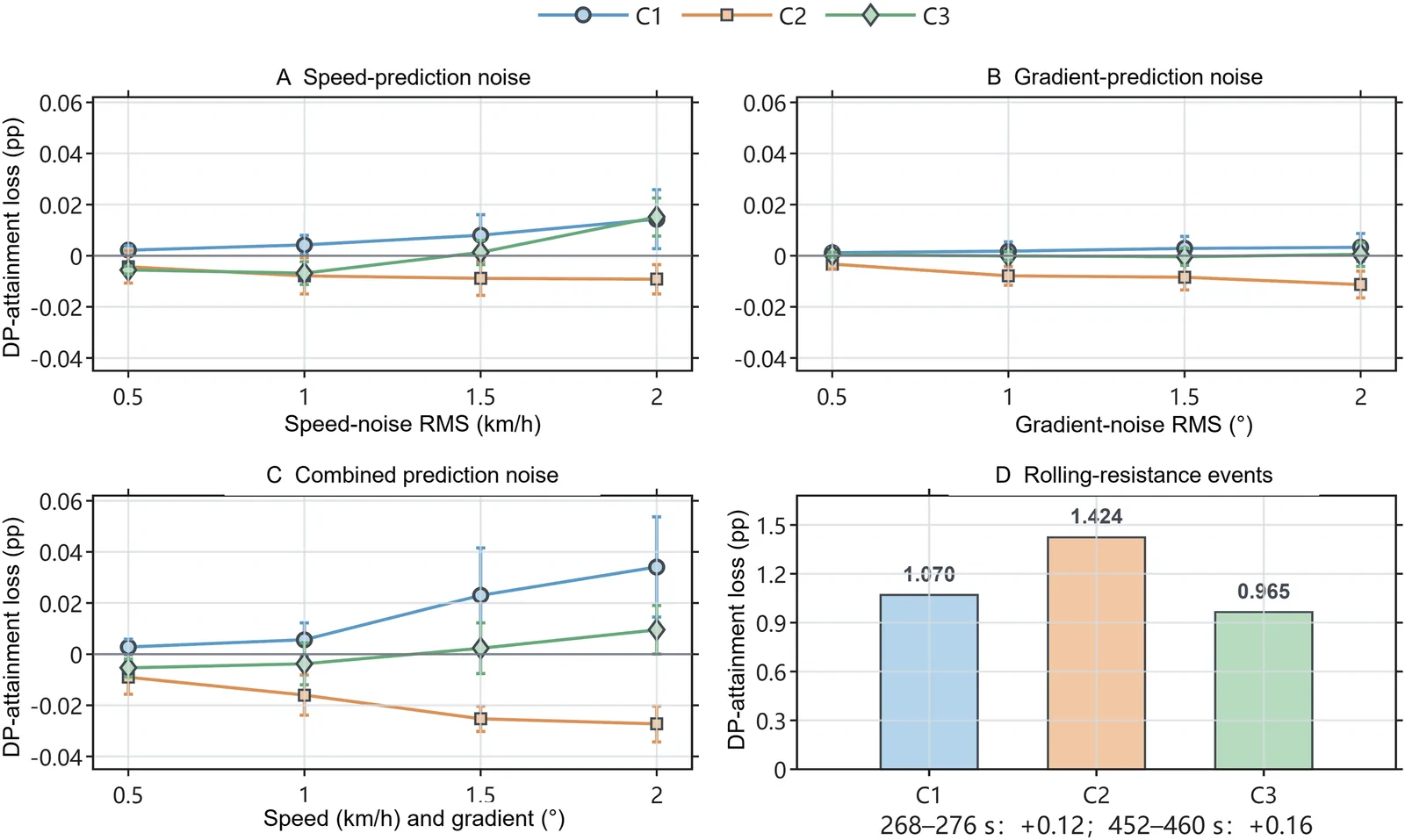
Robustness to prediction noise and abrupt rolling-resistance disturbances. Panels A–C show speed-only, gradient-only, and combined prediction-noise tests with 10 random seeds per level. Panel D shows attainment losses caused by the two equivalent rolling-resistance increments.

To examine the prediction-error–risk–torque propagation path, predicted inputs and all three power-source torques were recorded, and load risk was reconstructed at every sample using the controller equations. The same error levels were used, with 10 random-phase repetitions at the highest combined-noise level (Table 8). No sample exceeded the theoretical risk-error bound. At the highest error level, RMS risk changes did not exceed 0.044, torque changes remained small, and mode or ratio differences occupied only a limited fraction of the cycle. The reported absolute DP-attainment changes were below 0.035 percentage points. Small negative changes in C2 arose from local shifts in hybrid switching times and the SOC correction, rather than evidence that prediction errors improved performance. The results characterize bounded error propagation at both the supervisory-indicator and actuator-command levels.

**Table 8:** Propagation sensitivity at the highest combined prediction-error level.

Cycle	Risk change RMS	$\Delta T_{e}$ RMS (N·m)	$\Delta T_{m1}$ RMS (N·m)	$\Delta T_{m2}$ RMS (N·m)	Mode/ratio difference (%)	$\Delta R_{DP}$ (pp)
C1	0.0397	0.182	0.051	0.286	1.152/2.360	0.0341±0.0196
C2	0.0439	0.269	0.075	0.231	5.342/0.874	−0.0273±0.0070
C3	0.0429	0.204	0.057	0.195	4.431/0.662	0.0095±0.0095

*Note:* pp, percentage points. Ten random-phase repetitions were performed at the highest combined noise level. Mode/ratio entries report the two fractions separately.

Predictor parameters were then tested by one-factor-at-a-time variation around *N* = 10, *h* = 5 s, σ=4, and ρ=0.86. The ranges were N∈{6,8,10,12,14}, h∈{3,4,5,6,7} s, σ∈{2,3,4,5,6}, and ρ∈{0.78,0.82,0.86,0.90,0.94}. All 17 settings were simulated for 600 s in each of C1–C3. Except for the parameter under examination, the model, controller, cycle inputs, and SOC-correction method were unchanged. Bounded load-risk prediction RMSE and DP attainment were recorded (Fig 16).

**Fig 16:**
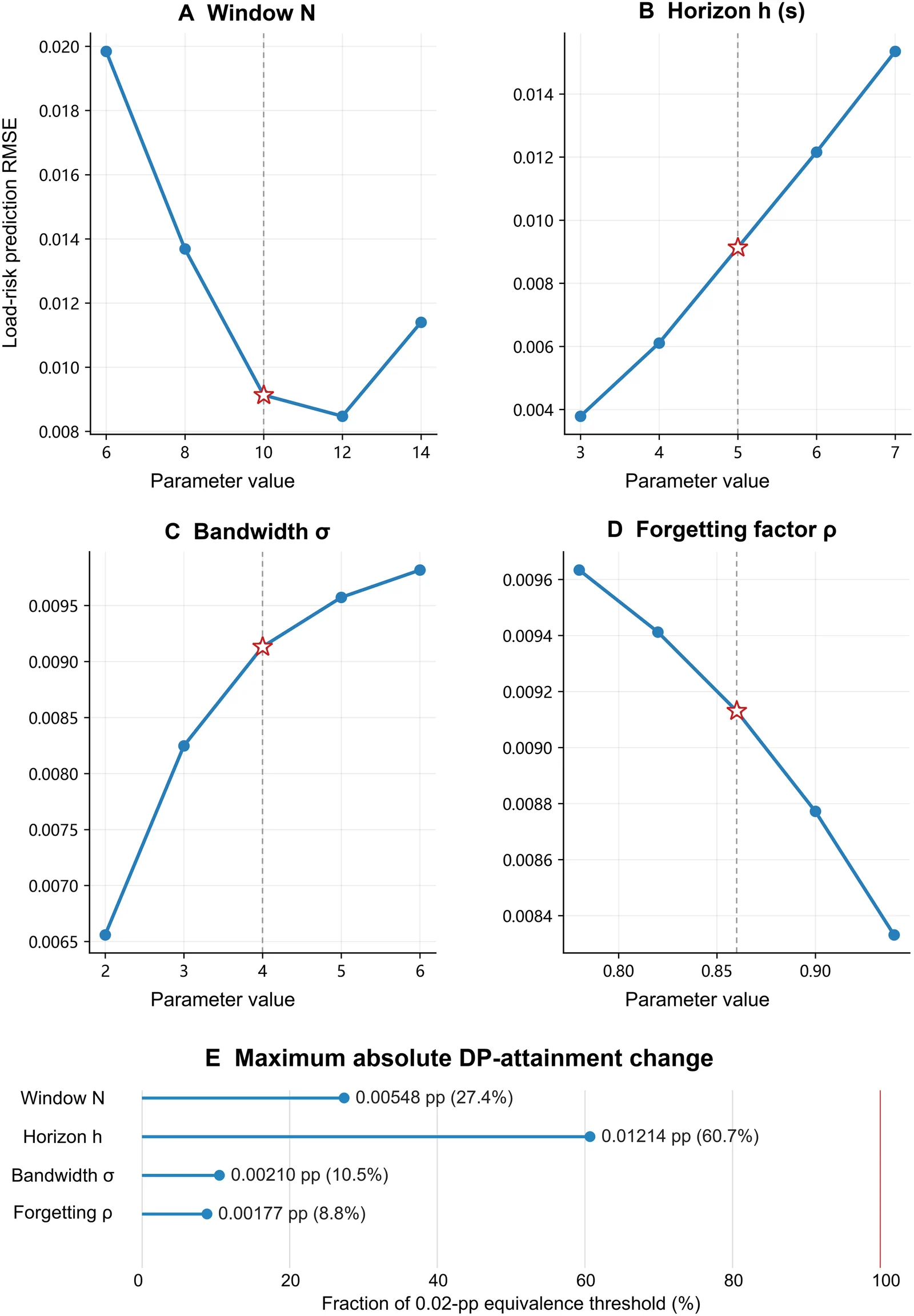
Predictor sensitivity and engineering equivalence of DP performance. Panels A–D show risk-prediction RMSE for changes in window length, prediction horizon, Gaussian bandwidth, and forgetting factor. Red stars mark the nominal settings. Panel E compares the largest absolute attainment change with the prespecified 0.02-percentage-point equivalence threshold.

Longer prediction horizons increased prediction difficulty. As the historical window grew, RMSE first decreased and then increased. RMSE generally increased with Gaussian bandwidth and decreased with the forgetting factor. These parameters thus had identifiable effects on risk-prediction accuracy. The nominal settings balance preview length, historical coverage, smoothing, and computation and lie near the centers of the tested ranges.

The largest single-cycle $\left|\Delta R_{DP}\right|$ values associated with window length, horizon, bandwidth, and forgetting factor were 0.00548, 0.01214, 0.00210, and 0.00177 percentage points, respectively. These represented 27.4%, 60.7%, 10.5%, and 8.8% of the prespecified ±0.02-percentage-point engineering-equivalence threshold. Across 48 nonbaseline single-cycle tests, changes ranged from −0.01031 to 0.01214 percentage points; all 16 parameter comparisons across cycles remained within the equivalence interval. Although parameter changes affected prediction error, the resulting DP-performance changes did not exceed the specified engineering threshold.

### Power-source dynamic response

Power-source response during load transitions complements whole-cycle fuel-economy evaluation. Figs 17–19 show actual engine and MG2 torque and speed under FULL in C1–C3.

**Fig 17:**
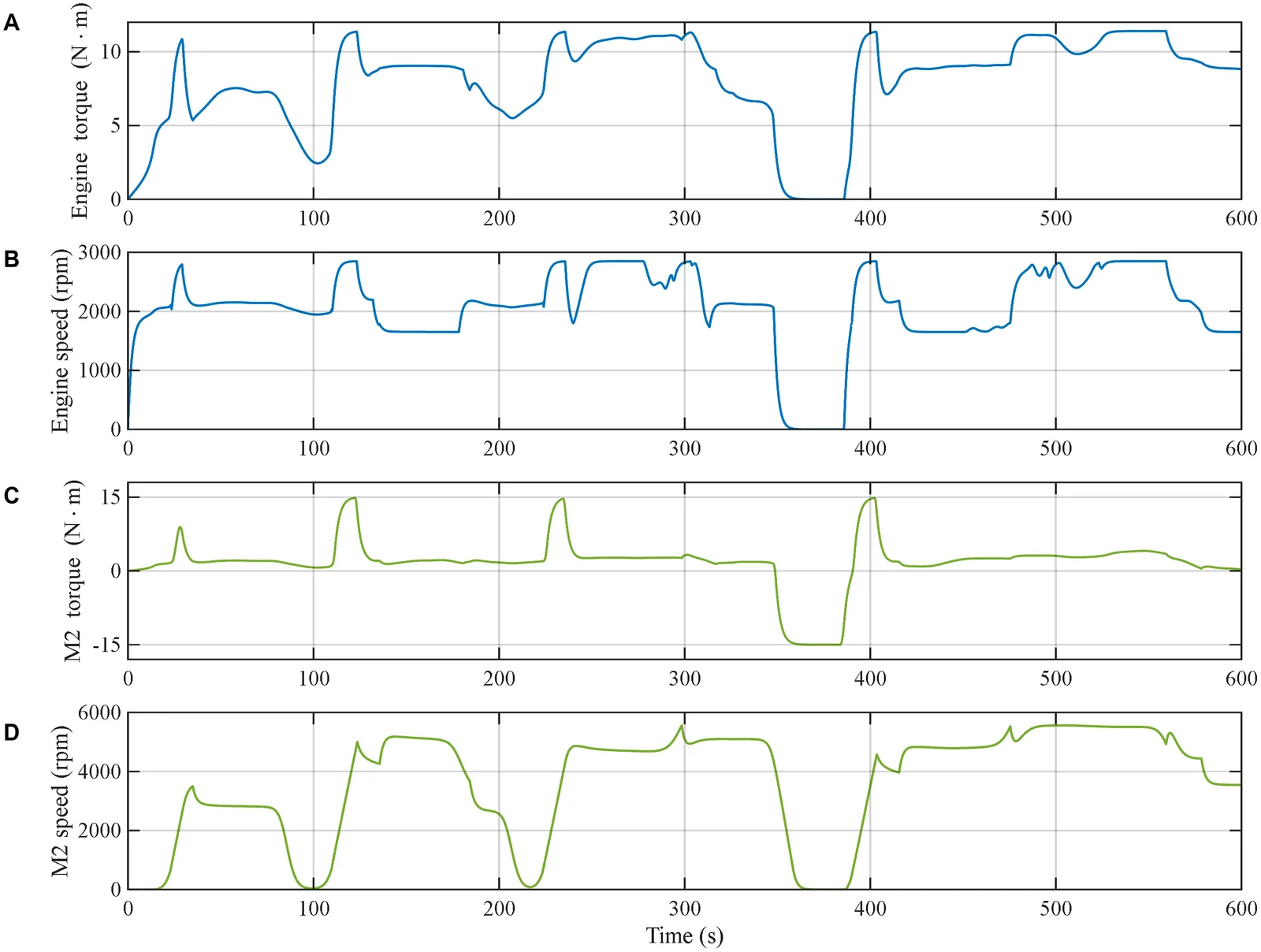
Engine and MG2 dynamic responses in C1 under FULL. Panels A–D show engine torque, engine speed, MG2 torque, and MG2 speed, respectively, during repeated starts and stops and coupled load changes.

**Fig 18:**
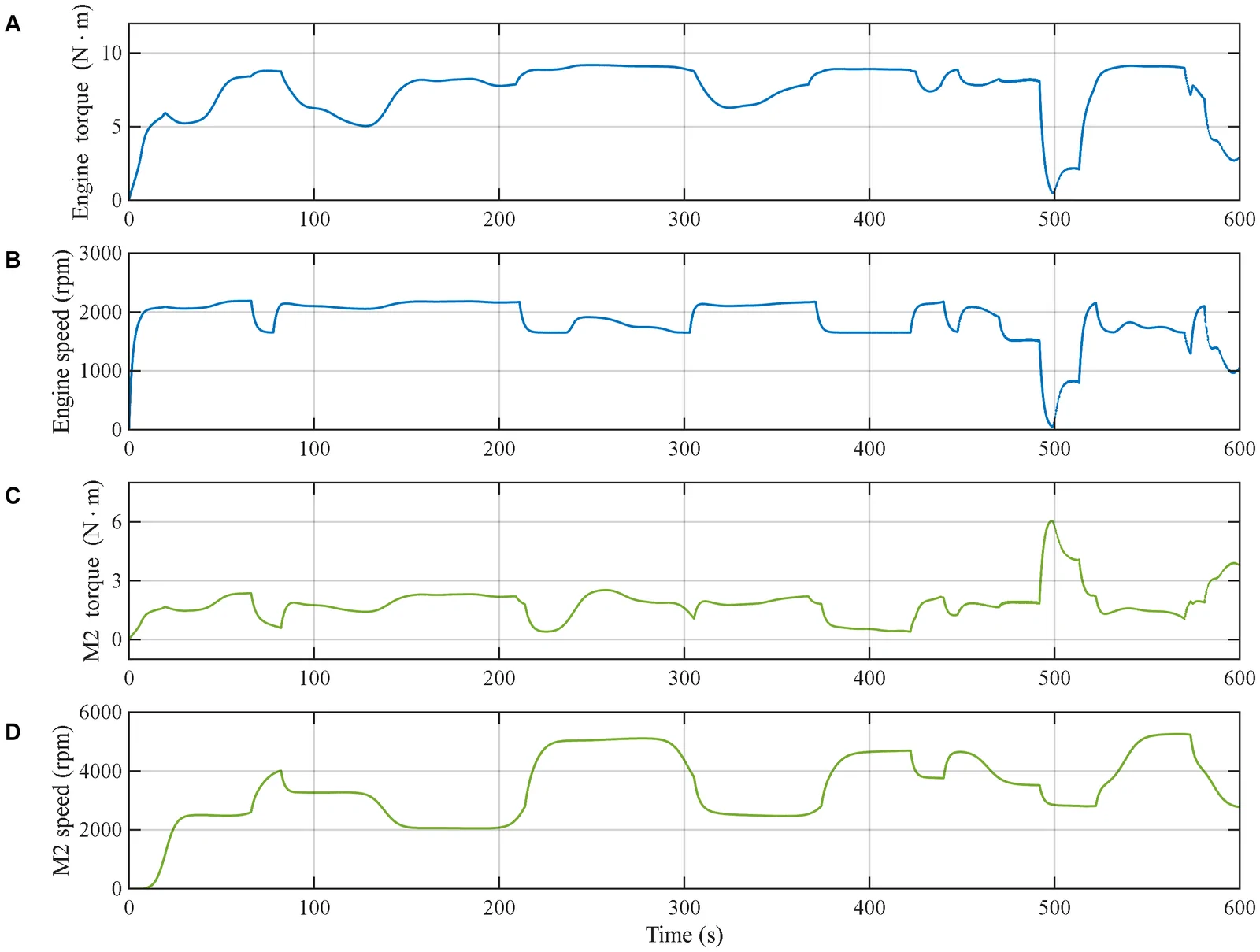
Engine and MG2 dynamic responses in C2 under FULL. Panels A–D show engine torque, engine speed, MG2 torque, and MG2 speed, respectively, during continuous travel with gradually varying gradient.

**Fig 19:**
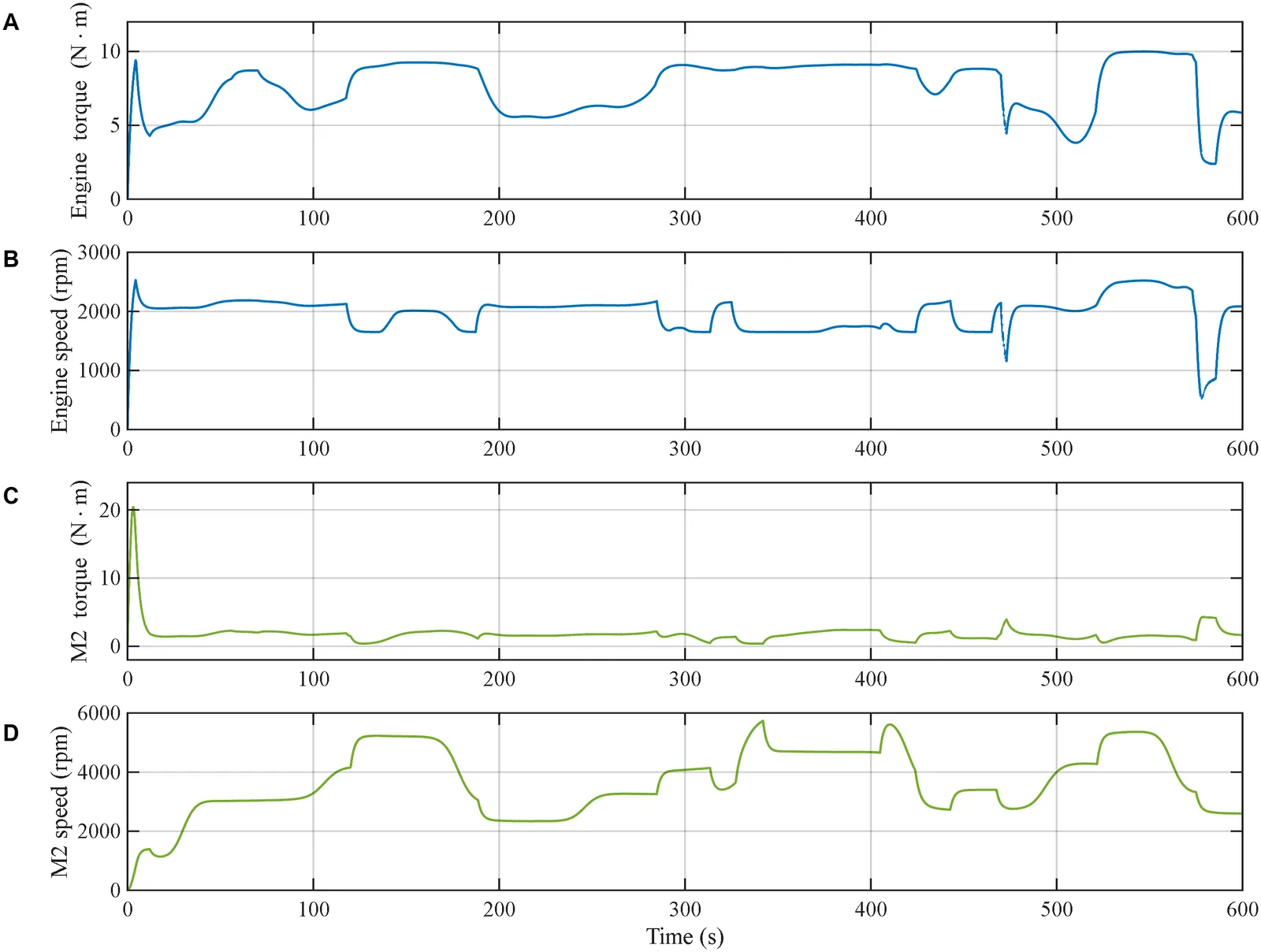
Engine and MG2 dynamic responses in C3 under FULL. Panels A–D show engine torque, engine speed, MG2 torque, and MG2 speed, respectively, during speed steps and composite gradient changes.

In C1, engine torque varied between 0 and 11.4 N·m with load; engine torque and speed fell to zero during stops. MG2 supplied brief positive torque at launch and during load increases and negative torque during deceleration at approximately 350–390 s. In C2, the engine operated mostly at approximately 5–9 N·m and 1600–2200 rpm. MG2 primarily supplied 0–3 N·m to smooth the load, with brief increases near 500 s and near the end of the cycle. In C3, the engine operating point shifted between stages. Apart from an approximately 20-N·m launch peak, MG2 operated mostly at low torque. Overall, the engine supplied sustained power, while MG2 supported launch, transient compensation, and braking-energy regulation.

### Vehicle experiments

The controller was evaluated on a laboratory tracked hybrid agricultural machine using same-route comparative tests (Fig 20). The vehicle operated without an attached implement on a maintained, firm mountainous lane under the rule-based controller and FULL. Travel distance was 571.4 m and mean speed was 3.43 km/h. Vehicle mass, route, initial SOC, warm-up condition, fuel-metering method, and speed-tracking requirements were held consistent between the tests. Cumulative fuel consumption, SOC, and speed were recorded simultaneously.

**Fig 20:**
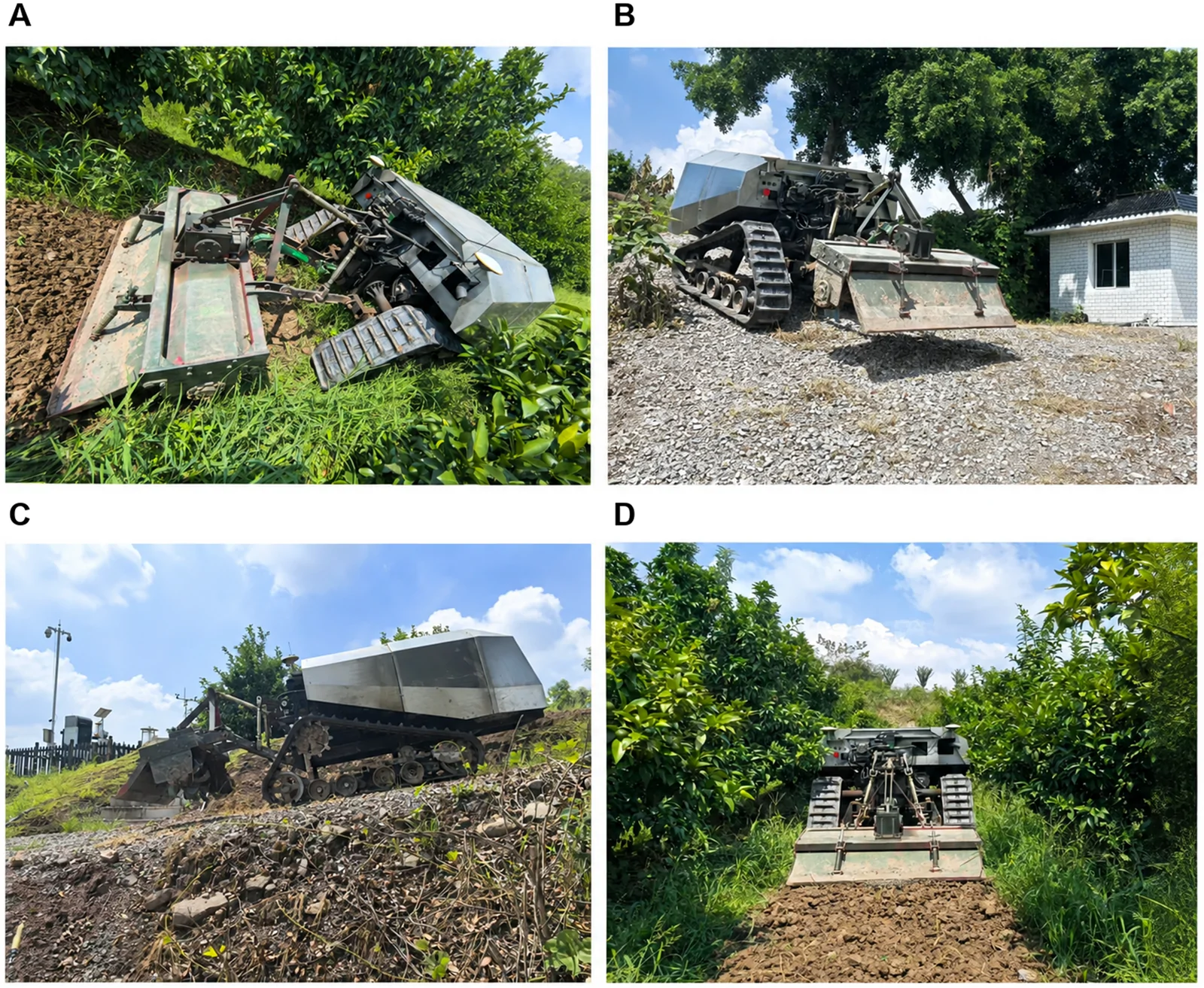
Tracked hybrid agricultural machine used for vehicle experiments. Panels A–D show four views of the platform. All photographs were taken by Xianfeng Zhang on 21 July 2026 and are distributed under the Creative Commons Attribution 4.0 International (CC BY 4.0) license.

Because terminal SOC differed between strategies, actual fuel consumption was reported together with SOC-corrected consumption using Eq (34) and the same coefficient, $K_{SOC}=5257.355$ g/SOC. Table 9 summarizes the results.

**Table 9:** Fuel consumption and SOC in the vehicle experiments.

Strategy	Actual fuel (g)	Initial SOC	Terminal SOC	Corrected fuel (g)	Fuel reduction (%)
Rule based	273.115	0.600000	0.597120	288.256	0
FULL	264.537	0.600000	0.598537	272.229	5.56

Actual fuel consumption decreased from 273.115 g with the rule-based controller to 264.537 g with FULL, while terminal SOC increased from 0.597120 to 0.598537. After correction to a common terminal-energy basis, fuel consumption was 288.256 and 272.229 g, respectively, a 5.56% reduction for FULL. The magnitude of the measured improvement differed from the simulation results, but fuel consumption and SOC retention changed in the same direction. These tests provide preliminary experimental evidence of controller implementability and energy-saving potential.

## Conclusions and outlook

### Main findings

A forward model and predictive-feedforward energy-management strategy were developed for a dual-motor power-split hybrid agricultural machine operating under coupled gradient, rolling-resistance, and power-source dynamics. The strategy combines short-horizon speed and gradient prediction, bounded load-risk feedforward, dynamic SOC safety boundaries, and transmission-ratio coordination. Offline DP supplied a common reference for evaluation across cycles.

Within the C1–C3 range of speeds below 10 km/h, gradients from $-2.29^{\circ }$ to $13.78^{\circ }$, and rolling-resistance coefficients from 0.100 to 0.151, the forward model mapped inertial, gradient, and time-varying rolling loads to wheel-end demand. Planetary kinematics and power balance described the resulting shifts in power-source operating points. This physical chain linked terrain disturbances, transmission constraints, and energy flow, providing a basis for mode selection and power allocation.

The bounded load-risk indicator established a supervisory link between prediction and execution. Predictions were normalized, weighted, and saturated before adjusting SOC boundaries, operating modes, and ratio thresholds. This limited their direct role in torque calculation and the gain of error propagation to power-source commands. FULL attained 93.29–95.59% of the DP reference performance. Removing both predictive channels and their associated coordination parameters reduced attainment by 9.13, 6.29, and 11.24 percentage points in C1–C3, showing the combined contribution of prediction and state, power-source, and ratio coordination.

The hierarchy separated load-risk representation, state-boundary adjustment, mode and ratio selection, and torque allocation. Physical constraints and structured rules reduced online search while retaining preview information. No SOC, battery-current, or power-source torque constraint was violated at the four tested prediction-noise levels or under the abrupt rolling-resistance increments. The largest rolling-disturbance-induced attainment loss was 1.4239 percentage points. Mean per-step execution time was 0.128–0.191 ms, 93.54% below that of MPC. These results quantify the balance achieved among computational demand, disturbance response, and control performance.

### Limitations and future work

The study primarily evaluated the tracked hybrid architecture and its energy management through simulation, supplemented by preliminary vehicle tests. Three limitations remain.

First, energy allocation addressed vehicle propulsion; the power demands of mowing, rotary tillage, transport implements, and other attachments were not included in a common controller. Future work could combine implement identification, load prediction, and coordinated propulsion–implement power allocation. This would allow the engine, electric machines, battery, and working actuators to respond jointly to implement blockage, impact loads, or abrupt traction demand while maintaining operation and protecting components.

Second, the representative cycles mainly described longitudinal travel with gradual changes in gradient. Sustained steep slopes, abrupt changes in gradient, transitions between longitudinal and cross slopes, and intermittent starts and stops were not covered comprehensively. Grade-compensation capability under steep conditions was also not assessed systematically. The conclusions therefore apply mainly to orchard lanes with relatively gradual gradient changes; stability and robustness on extreme and more complex slopes require further validation.

Third, the objectives addressed fuel economy, SOC retention, and computational performance. Battery temperature and aging, engine thermal state, motor temperature rise, and component lifetime were not considered jointly. These factors require further study through coupled physical models and multi-objective control.

## Supporting information

S1 File Tabulated study data and engine and electric-machine map data. The archive Minimum packet size.zip contains three Excel workbooks in the Minimum packet size folder. The workbook S1_Dataset.xlsx contains eight worksheets corresponding to Tables 1 and 3–9: “Table 1 Parameters” lists vehicle and component parameters and their units; “Table 3 Cycles” summarizes the duration, speed, stopped-time fraction, gradient ranges, and rolling-resistance ranges of cycles C1–C3; “Table 4 DP” reports fuel consumption and initial and terminal SOC for the offline DP benchmark; “Table 5 FULL” reports fuel consumption, SOC balance, and DP attainment for FULL; “Table 6 Strategies” compares FULL with the online baseline strategies; “Table 7 Ablation” reports corrected fuel consumption, terminal SOC, DP attainment, and attainment losses for the control-module ablations; “Table 8 Error Propagation” reports risk and torque root-mean-square changes, mode and ratio differences, and the mean and standard deviation of DP-attainment changes at the highest combined prediction-error level; and “Table 9 Vehicle Tests” reports actual and SOC-corrected fuel consumption, initial and terminal SOC, and fuel reduction in the vehicle experiments. The workbook Electric Motormap.xlsx, in the Electric Motormap folder, provides the engine speed–torque–specific-fuel-consumption data and full-load speed–torque curve associated with Fig 3. The workbook Motor Map.xlsx, in the Motormap folder, provides the electric-machine efficiency grid indexed by speed and torque associated with Fig 4.
(ZIP)
